# Supplementary material for: Diagnostic yield of genetic screening in a diverse, community-ascertained cohort
Source: Genome Med. 2023 Apr 18;15:26. doi: 10.1186/s13073-023-01174-7 (PMC10111761; doi:10.1186/s13073-023-01174-7)
Supplement: Supplementary file 1 — Additional file 1: Figure S1. Study protocol for population genetic screening. Table S1. Detailed demographics of enrollees. Table S2. Study participation at different stages of population genetic screening by race and ethnicity. Table S3. Association of race and ethnicity with enrollment. Table S4. Probability of screening enrollment for men and women aged 40 in different race and ethnicity groups. Table S5. Variants detected in population genetic screening study. Supplemental Methods. (1) Screening assay design and validation, (2) Data analysis, (3) Initial recruitment email, (4) Study information and FAQ, (5) Summary of survey instruments and measures, (6) T0 survey, (7) T1 survey, (8) T2 survey, (9) Example uninformative results letter, (10) Example positive results letter. [file 13073_2023_1174_MOESM1_ESM.docx]

**Additional File 1**

Table of Contents

[Figure S1: Study protocol for population genetic screening 2](#_Toc126691987)

[Table S1: Detailed demographics of enrollees 3](#_Toc126691988)

[Table S2: Study participation at different stages of population genetic screening by race and ethnicity 5](#_Toc126691989)

[Table S3: Association of race and ethnicity with enrollment 5](#_Toc126691990)

[Table S4: Probability of screening enrollment for men and women aged 40 in race and ethnicity groups where a significant interaction was present with gender and race and ethnicity 5](#_Toc126691991)

[Table S5: Variants detected in the population genetic screening study 6](#_Toc126691992)

[Supplemental Methods 9](#_Toc126691993)

[1. Screening assay design and validation 9](#_Toc126691994)

[2. Data analysis 10](#_Toc126691995)

[3. Initial recruitment email 13](#_Toc126691996)

[4. Study information and frequently asked questions provided by the population genetic screening study at UWM 14](#_Toc126691997)

[5. Summary of survey instruments and measures 16](#_Toc126691998)

[6. T0 Survey 17](#_Toc126691999)

[7. T1 Survey 20](#_Toc126692000)

[8. T2 Survey 47](#_Toc126692001)

[9. Example uninformative results letter 52](#_Toc126692002)

[10. Example positive results letter 53](#_Toc126692003)

# **Figure S1: Study protocol for population genetic screening**

Email invitation

Clicked email link to FAQ

Interested in continuing?

Yes

No

T0 Survey

Open text box to provide reason(s) for declining

Interested in receiving DNA kit?

Provide address info

Open text box to provide reason(s) for declining

Yes

No

DNA kits & consent forms sent

DNA kits & consent forms returned

T1 Survey

Result return

T2 Survey

Yes

No

# **Table S1: Detailed demographics of enrollees**

| **Characteristic** | **N (%)** |
| --- | --- |
| **Adopted** |  |
| Yes | 115 (4.0) |
| No | 2762 (95.6) |
| Missing | 12 (0.4) |
| **Personal cancer diagnosis** |  |
| Yes | 321 (11.1) |
| No | 2560 (88.6) |
| Missing | 8 (0.3) |
| **Family cancer diagnosis**^a^ |  |
| Yes | 1535 (53.2) |
| No | 1024 (35.4) |
| Don’t know | 318 (11.0) |
| Missing | 12 (0.4) |
| **Personal heart attack**^b^ |  |
| Yes | 43 (1.5) |
| No | 2835 (98.1) |
| Missing | 11 (0.4) |
| **Family heart attack**^a^ |  |
| Yes | 1010 (34.9) |
| No | 1327 (46.0) |
| Don’t know | 545 (18.9) |
| Missing | 7 (0.2) |
| **Sex assigned at birth** |  |
| Male | 765 (26.4) |
| Female | 1295 (44.9) |
| Other | 0 (0) |
| Prefer not to answer | 3 (0.1) |
| Missing | 826 (28.6) |
| **Sexual orientation**^c^ |  |
| Asexual | 84 (2.9) |
| Bisexual | 147 (5.1) |
| Gay or lesbian | 172 (6.0) |
| Queer | 77 (2.7) |
| Straight | 1560 (54.0) |
| Something else | 20 (0.7) |
| Don’t know | 10 (0.4) |
| Prefer not to answer | 42 (1.5) |
| Missing | 841 (29.1) |
| **Education** |  |
| Less than high school | 6 (0.2) |
| High school/GED | 61 (2.1) |
| Some college | 299 (10.4) |
| College graduate | 865 (29.9) |
| Advanced degree | 887 (30.7) |
| Missing | 771 (26.7) |
| **Household Income** |  |
| <$50,000 | 304 (10.5) |
| >$50,000 but ≤100,000 | 539 (18.7) |
| >$100,000 | 1105 (38.2) |
| Prefer not to answer | 190 (6.6) |
| Missing | 751 (26.0) |
| **Past genetic testing** |  |
| Yes | 425 (14.7) |
| No | 1689 (58.5) |
| Missing | 775 (26.8) |
| **Family genetic testing showing increased risk**^d^ |  |
| Yes | 99 (3.4) |
| No | 1959 (67.8) |
| Missing | 831 (28.8) |

*^a^*Family was defined as a close biological relative (mother, father, son, daughter, aunt, uncle)

*^b^*At any point in time

*^c^*Multiple choices could be selected

*^d^*Degree of relatedness was not specified in question. This question was asked to all people administered the T1 survey and a not applicable option to indicate a lack of family genetic testing was not provided

**Table S2: Study participation at different stages of population genetic screening by race and ethnicity** [N (%)]

|  | **Overall** | **African American** | **Asian** | **Hispanic** | **Other** | **Native American** | **White** | **Missing** |
| --- | --- | --- | --- | --- | --- | --- | --- | --- |
| **Invited** | 40857 (100) | 10267 (100) | 21707 (100) | 4099 (100) | 169 (100) | 1533 (100) | 3063 (100) | 19  (100) |
| **Accessed FAQ** | 5231 (12.8) | 784  (7.6) | 2801 (12.9) | 644  (15.7) | 54 (32.0) | 238  (15.5) | 692 (22.6) | 18 (94.7) |
| **Requested kit** | 4528 (11.08) | 634  (6.18) | 2413 (11.12) | 573 (13.98) | 48 (28.4) | 213 (13.89) | 629 (20.54) | 18 (94.7) |
| **Sent kit** | 4502 (11.02) | 630  (6.14) | 2399 (11.05) | 570 (13.91) | 46 (27.2) | 212 (13.83) | 627 (20.47) | 18 (94.7) |
| **Sample & signed consent returned** | 2889 (7.1) | 333  (3.2) | 1625 (7.5) | 369  (9) | 23 (13.6) | 99  (6.5) | 427 (13.9) | 13 (68.4) |

# **Table S3: Association of race and ethnicity with enrollment**

| **Race/Ethnicity** | **Beta** | **SE** | **P-Value** | **OR (95% CI)** |
| --- | --- | --- | --- | --- |
| African American | -0.90 | 0.06 | <2e-16 | 0.41 (0.36, 0.46) |
| Hispanic | 0.19 | 0.06 | 0.002 | 1.21 (1.07, 1.36) |
| Multiracial/Other | 0.56 | 0.24 | 0.018 | 1.75 (1.1, 2.79) |
| Native American | -0.19 | 0.11 | 0.072 | 0.82 (0.67, 1.02) |
| White | 0.59 | 0.06 | <2e-16 | 1.81 (1.61, 2.04) |

*adjusted for age (continuous) and gender (male, female, other); Asian as reference

# **Table S4: Probability of screening enrollment for men and women aged 40 in race and ethnicity groups where a significant interaction was present with gender and race and ethnicity**

|  | **African American** | **Asian** | **Hispanic** |
| --- | --- | --- | --- |
| **Female** | 0.04 | 0.08 | 0.11 |
| **Male** | 0.03 | 0.07 | 0.07 |

Asian as reference

# **Table S5: Variants detected in the population genetic screening study**

| **Study ID** | **Gene and variant** | **Known to enrollee (Yes/No)** | **Known in first degree relative (Yes/No)** | **gnomAD**  **v2.1.1**  **count** | **ClinVar ID** |
| --- | --- | --- | --- | --- | --- |
| 36534 | APC NM_000038.5:c.221-2A>G | Y | N | 1 | 184117 |
| 14454 | APC NM_000038.5:c.3920T>A, p.Ile1307Lys | N | N | 524 | 822 |
| 44440 | APC NM_000038.5:c.3920T>A, p.Ile1307Lys | N | N | 524 | 822 |
| 32321 | APOB NM_000384.3:c.10580G>A, p.Arg3527Gln | N | N | 83 | 17890 |
| 36032 | APOB NM_000384.3:c.10580G>A, p.Arg3527Gln | N | N | 83 | 17890 |
| 5023 | ATM NM_000051.3:c.1564_1565del, p.Glu522Ilefs*43 | N | N | 14 | 127340 |
| 2032 | ATM NM_000051.3:c.1939G>T, p.Glu647* | Y | N | 0 | 407556 |
| 4694 | ATM NM_000051.3:c.3372C>G, p.Tyr1124* | N | N | 1 | 127371 |
| 36465 | ATM NM_000051.3:c.5763-1050A>G | N | N | 5 | 3021 |
| 23715 | ATM NM_000051.3:c.6347+1G>A | N | N | 1 | 371243 |
| 18270 | ATM NM_000051.3:c.742C>T, p.Arg248* | N | N | 3 | 181913 |
| 21727 | ATM NM_000051.3:c.8786+1G>A | N | N | 4 | 127463 |
| 18588 | BRCA1 NM_007294.3 exon 9-12 del | Y | N | NA | - |
| 22110 | BRCA1 NM_007294.3:c.181T>G, p.Cys61Gly | Y | N | 8 | 54360 |
| 44396 | BRCA1 NM_007294.3:c.188T>A, p.Lys63* | N | N | 0 | 54381 |
| 38565 | BRCA1 NM_007294.3:c.191G>A, p.Cys64Tyr | N | N | 0 | 54400 |
| 3023 | BRCA1 NM_007294.3:c.1960A>T, p.Lys654* | Y | N | 1 | 37436 |
| 23736 | BRCA1 NM_007294.3:c.3756_3759del, p.Ser1253Argfs*10 | N | N | 6 | 17673 |
| 15348 | BRCA1 NM_007294.3:c.5359_5363delinsAGTGA, p.Cys1787_Gly1788delinsSerAsp | Y | N | 0 | 187349 |
| 26291 | BRCA1 NM_007294.3:c.5470_5477del, p.Ile1824Aspfs*3 | N | N | 0 | 267222 |
| 1037 | BRCA2 NM_000059.3:c.1796_1800del, p.Ser599* | Y | N | 1 | 37756 |
| 32904 | BRCA2 NM_000059.3:c.2059_2063del, p.Asp687* | N | N | 1 | 142868 |
| 256 | BRCA2 NM_000059.3:c.2677del, p.Gln893Lysfs*2 | Y | N | 0 | 246127 |
| 35852 | BRCA2 NM_000059.3:c.316+1G>A | Y | N | 0 | 37822 |
| 2961 | BRCA2 NM_000059.3:c.3264dup, p.Gln1089Serfs*10 | Y | N | 6 | 37830 |
| 747 | BRCA2 NM_000059.3:c.3554_3555del, p.Thr1185Serfs*2 | Y | N | 0 | 51485 |
| 26195 | BRCA2 NM_000059.3:c.3860del, p.Asn1287Ilefs*6 | N | N | 12 | 51545 |
| 20449 | BRCA2 NM_000059.3:c.4111C>T, p.Gln1371* | Y | N | 0 | 51599 |
| 28913 | BRCA2 NM_000059.3:c.4449del, p.Asp1484Thrfs*2 | Y | N | 0 | 37904 |
| 3696 | BRCA2 NM_000059.3:c.4588A>T, p.Lys1530* | N | N | 0 | 51678 |
| 2087 | BRCA2 NM_000059.3:c.4631del, p.Asn1544Thrfs*24 | N | N | 1 | 37913 |
| 35449 | BRCA2 NM_000059.3:c.5238dup, p.Asn1747* | N | N | 0 | 37954 |
| 147 | BRCA2 NM_000059.3:c.5454del, p.Cys1820fs | Y | N | 0 | 51864 |
| 37902 | BRCA2 NM_000059.3:c.5909C>A, p.Ser1970* | N | N | 0 | 38007 |
| 1312 | BRCA2 NM_000059.3:c.6275_6276del, p.L2092Pfs*7 | Y | N | 10 | 9318 |
| 18549 | BRCA2 NM_000059.3:c.6405_6409del, p.Asn2135Lysfs*3 | N | N | 1 | 38043 |
| 36547 | BRCA2 NM_000059.3:c.658_659del, p.Val220Ilefs*4 | N | N | 13 | 9342 |
| 2863 | BRCA2 NM_000059.3:c.6833_6837del, p.Ile2278fs | Y | N | 0 | 52203 |
| 20886 | BRCA2 NM_000059.3:c.6952C>T, p.Arg2318* | Y | N | 0 | 38076 |
| 43181 | BRCA2 NM_000059.3:c.7480C>T, p.Arg2494* | N | N | 8 | 38099 |
| 30333 | BRCA2 NM_000059.3:c.7588C>T, p.Gln2530* | N | Y | 0 | - |
| 1392 | BRCA2 NM_000059.3:c.771_775del, p.N257Kfs*17 | Y | N | 2 | 9326 |
| 20455 | BRCA2 NM_000059.3:c.8548_8551del, p.Glu2850Glnfs*12 | Y | Y | 0 | 38167 |
| 2032 | BRIP1 NM_032043.2:c.2253_2254del, p.Lys752Argfs*12 | N | N | 3 | 628733 |
| 48460 | BRIP1 NM_032043.2:c.2990_2993del, p.Thr997Argfs*61 | Y | N | 5 | 234281 |
| 32796 | BRIP1 NM_032043.2:c.3201C>A, p.Cys1067* | N | N | 0 | - |
| 1983 | BRIP1 NM_032043.2:c.985C>T, p.Gln329* | N | N | 0 | 936751 |
| 1164 | CDH1 NM_004360.3:c.1118dup, p.Ile374Aspfs*13, | N | N | 0 | - |
| 2892 | CHEK2 NM_007194.3:c.1100del, p.Thr367Metfs*15 | Y | N | 591 | 128042 |
| 30137 | CHEK2 NM_007194.3:c.1100del, p.Thr367Metfs*15 | N | N | 591 | 128042 |
| 32027 | CHEK2 NM_007194.3:c.1100del, p.Thr367Metfs*15 | N | N | 591 | 128042 |
| 24950 | CHEK2 NM_007194.3:c.219_223del, p.Ile74* | N | N | 2 | 460817 |
| 27967 | CHEK2 NM_007194.3:c.349A>G, p.Arg117Gly | N | N | 32 | 128071 |
| 3740 | CHEK2 NM_007194.3:c.470T>C p.Ile157Thr | N | N | 1391 | 5591 |
| 2863 | CHEK2 NM_007194.3:c.470T>C, p.Ile157Thr | N | N | 1391 | 5591 |
| 6635 | CHEK2 NM_007194.3:c.470T>C, p.Ile157Thr | N | N | 1391 | 5591 |
| 21650 | CHEK2 NM_007194.3:c.470T>C, p.Ile157Thr | N | Y | 1391 | 5591 |
| 29897 | CHEK2 NM_007194.3:c.470T>C, p.Ile157Thr | N | N | 1391 | 5591 |
| 32415 | CHEK2 NM_007194.3:c.846+4del | N | N | 0 | 838872 |
| 29954 | CHEK2 NM_007194.3:c.902del: p.Leu301Trpfs*3 | N | N | 1 | 185097 |
| 13189 | HOXB13 NM_006361.6:c.251G>A, p.Gly84Glu | N | N | 528 | 128031 |
| 16751 | HOXB13 NM_006361.6:c.251G>A, p.Gly84Glu | N | N | 528 | 128031 |
| 40102 | HOXB13 NM_006361.6:c.251G>A, p.Gly84Glu | N | N | 528 | 128031 |
| 36883 | LDLR NM_000527.4: exon 9-12 del | N | N | NA | - |
| 16025 | LDLR NM_000527.4:c.1247G>A, p.Arg416Gln | N | N | 5 | 251752 |
| 26472 | LDLR NM_000527.4:c.1424C>T, p.Ala475Val | N | N | 0 | 251836 |
| 21330 | LDLR NM_000527.4:c.1426C>T, p.Pro476Ser | N | N | 0 | 251837 |
| 34221 | LDLR NM_000527.4:c.1586+5G>A | N | N | 8 | 251909 |
| 11952 | LDLR NM_000527.4:c.1747C>T, p.His583Tyr | Y | N | 29 | 200921 |
| 35449 | LDLR NM_000527.4:c.1747C>T, p.His583Tyr | N | N | 29 | 200921 |
| 41409 | LDLR NM_000527.4:c.1747C>T, p.His583Tyr | N | N | 29 | 200921 |
| 45447 | LDLR NM_000527.4:c.1783C>T, p.Arg595Trp | N | N | 2 | 161290 |
| 16068 | LDLR NM_000527.4:c.249del, p.Pro84Leufs*122 | N | N | 0 | - |
| 35110 | LDLR NM_000527.4:c.268G>A, p.Asp90Asn | N | N | 17 | 251105 |
| 5861 | MLH1 NM_000249.3 exon 16-19 del | Y | N | NA | 89865 |
| 2063 | MLH1 NM_000249.3:c.1409+1G>A | Y | N | 0 | 89718 |
| 510 | MLH1 NM_000249.3:c.245C>T, p.Thr82Ile | Y | N | 0 | 90118 |
| 27469 | MLH1 NM_000249.3:c.793C>T, p.Arg265Cys | N | N | 0 | 29654 |
| 13694 | MSH2 NM_000251.2:c.1835C>G, p.S612* | Y | N | 0 | 90799 |
| 176 | MSH2 NM_000251.2:c.942+3A>T | Y | N | 1 | 36580 |
| 5968 | MSH6 NM_000179.2:c.2759del, p.Lys920Argfs*25 | N | N | 0 | 428433 |
| 34682 | MSH6 NM_000179.2:c.3119_3120del, p.Phe1040* | N | N | 1 | 89339 |
| 42458 | MSH6 NM_000179.2:c.3259_3260insA, p.Pro1087Hisfs*6 | N | N | 0 | - |
| 3790 | MSH6 NM_000179.2:c.3261dup, p.Phe1088Leufs*5, | N | N | 1 | 89364 |
| 1646 | PALB2 NM_024675.3:c.2167_2168del, p.Met723Valfs*21 | Y | N | 16 | 136132 |
| 17611 | PALB2 NM_024675.3:c.2167_2168del, p.Met723Valfs*21 | Y | N | 16 | 136132 |
| 2679 | PALB2 NM_024675.3:c.226del, p. Ile76Tyrfs*101 | N | N | 1 | 142408 |
| 32148 | PALB2 NM_024675.3:c.3183_3184del, p.His1061Glnfs*5 | N | N | 0 | - |
| 19496 | PALB2 NM_024675.3:c.3244_3245del, p.Ser1082* | N | N | 0 | 265368 |
| 19818 | PALB2 NM_024675.3:c.599del, p.Leu200* | N | N | 1 | 141880 |
| 26231 | PALB2 NM_024675.3:c.751C>T, p.Gln251* | N | N | 0 | 126767 |
| 16116 | PMS2 NM_000535.6:c.2444C>T, p.Ser815Leu | N | N | 2 | 91343 |
| 960 | PMS2 NM_000535.6:c.2444C>T, p.Ser815Leu | Y | N | 2 | 91343 |
| 37647 | PMS2 NM_000535.6:c.538-2A>G | N | N | 3 | 411028 |
| 28464 | PMS2 NM_000535.6:c.614A>C, p.Gln205Pro | N | N | 2 | 91361 |
| 1849 | RAD51C NM_058216.3:c.394dup, p.Thr132Asnfs*23 | N | Y | 10 | 182845 |
| 30125 | RAD51C NM_058216.3:c.394dup, p.Thr132Asnfs*23 | Y | N | 10 | 182845 |
| 40187 | RAD51C NM_058216.3:c.394dup, p.Thr132Asnfs*23 | N | N | 10 | 182845 |
| 782 | RAD51C NM_058216.3:c.404+2T>C | N | N | 1 | 182835 |
| 16669 | RAD51C NM_058216.3:c.577C>T, p.Arg193* | N | N | 10 | 140849 |
| 19704 | RAD51C NM_058216.3:c.905-2A>C | N | N | 4 | 216132 |
| 36277 | RAD51C NM_058216.3:c.97C>T, p.Gln33* | N | N | 3 | 142534 |
| 23550 | RAD51D NM_002878.3:c.131_144+24del | N | N | 1 | 422338 |
| 27492 | RAD51D NM_002878.3:c.270_271dup, p.Lys91leIfs*13 | N | N | 14 | 239394 |
| 1380 | RAD51D NM_002878.3:c.694C>T, p.Arg232* | Y | N | 4 | 127893 |
| 43871 | STK11 NM_000455.4:c.862+3G>T | N | N | 0 | - |

**Supplemental Methods:**

## **Screening assay design and validation**

The screening panel described to invitees consisted of genes associated with diseases or disorders of cancer and high lipids, for which clinical procedures or therapies are available for disease prevention, mitigation, and treatment. The testing panel was developed by UWM, Genetics and Solid Tumors Laboratory to detect pathogenic and likely pathogenic variants in the 25 covered genes including variants at intron/exon boundaries and single or multi-exon deletions or duplications. A list of the targeted genes and their associated conditions was available on the study website. For two genes, *MUTYH* and *NTHL1*, it was decided to report only homozygous or compound heterozygous pathogenic and likely pathogenic variants.

DNA from Oragene saliva sample kits was isolated for sequencing at the UWM Genetics and Solid Tumors Laboratory or the UWM Northwest Clinical Genomics Laboratory using QIAsymphony automated extraction. DNA libraries were generated using IDT Lotus library prep kits followed by capture using a custom set of IDT biotinylated probes. Pools of between six and 188 enrollee samples with appropriate controls were sequenced on Illumina MiSeq or Next Seq sequencers. The bioinformatics pipeline for next-generation sequence analysis was a minimally-modified version of the clinical UW ColoSeq assay pipeline and performance was expected to mirror performance of the clinical BROCA assay.^1,2^ Several unique customizations were incorporated into the bioinformatic pipeline, including processes to detect copy number variants in all exons of *PMS2*.^3^

Prior to the screening study, the panel was validated using 124 samples with 129 known variants of interest together with 9,404 variants from well characterized biobank samples. Across all variant types, the panel had 98.4% sensitivity for variants of interest, with lower sensitivity for indels greater than 12 bp and complex copy number variants, and 99.98% specificity for covered variants in biobank samples.

## **Data analysis**

*Screening enrollment and drop out*

Data on the process of genetic screening collected in a REDCap database included number of invites sent, number of DNA sample collection kits sent, enrollees, failed samples, and repeat testing. Demographic data, including age, gender, race, and ethnicity, were available for all study invitees through the UWM EHR. The T1 survey also asked about gender, race, and ethnicity. As T1 survey data were not reported for all invitees, EHR demographics were used for analyses to maximize available data. However, individuals who responded “Prefer not to answer” for the gender question in the T1 survey were classified as such during analyses to respect potential desires to refrain from reporting gender.

We defined enrollment in this study as the return of DNA samples and signed consent forms. T0 characteristics of interest included if enrollees were adopted, had a personal diagnosis of cancer, a diagnosis of cancer in the family, had ever experienced a heart attack or had a family member experience a heart attack. Family in these questions was defined as a close biological relative, such as a mother, father, son, daughter, aunt or uncle. T1 characteristics of interest included sex assigned at birth, sexual orientation, education, income, any past genetic testing, or any genetic testing in the family indicating an increased disease risk. Similar to the T0 survey, the T1 survey also asked about personal and family history of cancer or heart attack and proceeded to go into more detail to learn exactly which family members had experienced these conditions. However, because T1 survey completion was lower compared to T0, T0 survey data were used to report personal and familial cancer and heart attack status.

We calculated the number of study invitees and enrollees and descriptive statistics for study invitees and enrollees using EHR demographic data. Demographics of enrollees were further described using data from the T0 and T1 surveys. We also descriptively assessed overall drop out in this screening study by looking at the number of people who proceeded through different steps: (1) sent study invites, (2) accessed study FAQ, (3) requested a DNA collection kit, (4) sent a DNA collection kit and consent forms, (5) returned DNA sample and signed consent forms, (6) sent results. For the sent results step, we described the number of people with available screening results. We additionally reported the number of people with positive results that the study genetic counselor was able to reach to discuss results. We also analyzed enrollee activity on the study results online portal to determine the number of enrollees with uninformative results who accessed their result letter. Study drop out was also descriptively assessed by race and ethnicity.

We used two logistic regression models to examine the association between race and ethnicity and enrollment in population genetic screening. Model A included age (linear), gender (Male, Female, Other), and race/ethnicity (ascertained from the EHR: Hispanic (of any race), Asian [reference], African American, Multiracial/Other, Native American, White (latter five categories were all Non-Hispanic) as covariates. Model B additionally included a gender by race and ethnicity interaction term as differences in engagement in genetic services and research has been seen based on gender and race and ethnicity.^4–6^ We investigated significant interaction effects by calculating the probability of enrollment for individuals in groups relevant to the interaction using the mean age of invitees. We also conducted an exploratory analysis to assess the relationship of sexual orientation (a binary variable indicating if an individual was in the group of 1,000 LGBTQ+ invitees as coded in the EHR) with study enrollment by adding sexual orientation and a sexual orientation by race and ethnicity interaction term as additional covariates to the original Model A. Individuals who had selected “Prefer not to answer” for questions regarding gender in the T1 survey were excluded from these analyses.

*Screening yield*

We described the total number of enrollees with screening results, including the number of positive and uninformative screening results and the pathogenic and likely pathogenic variants detected. Using T1 survey data, we calculated the number of people with positive results finding out about their genetic variant for the first time through screening. Comparisons of self-report of testing between groups receiving positive and uninformative results were performed using Fishers exact test.

*Assessment of personal and family history*

We separated enrollees into several categories according to self-reported personal and family history: those already aware of their variant, those who reported a first-degree relative with a variant, those with a personal diagnosis of disease, those with a first degree relative diagnosed with disease, and those with no reported family history of disease. The T0 pre-enrollment survey only asked a few high-level questions about personal and family history of cancer and heart disease. The T1 survey asked more detailed questions about personal and family history. For family history assessment, data from the T1 survey were used when possible.

*Assessment of guidelines*

We used self-reported personal and family history, including history of prior genetic testing to determine if the enrollees were likely to have qualified for genetic testing under National Comprehensive Cancer Network (NCCN) guidelines for any of the cancer-risk genes on the panel or if they would have qualified for familial hypercholesterolemia testing under American College of Cardiology guidelines.^7,8^ Because personal and family history were self-reported and the study did not have access to complete medical records, it was only possible to evaluate a few of the guideline criteria. Most criteria for familial hypercholesterolemia diagnosis rely on lipid measures, which were not available to the study.

## **Initial recruitment email**

| Subject: University of Washington Genetic Screening for Inherited Disease Risk Study  Dear [First Name Here],  Because you have received health care from the University of Washington Medical Center or UW clinics, we are asking you if you will join us in a new research study.  The study will use new genetic technology to help people learn if their genes increase their risk for developing certain diseases like heart disease and cancer.  Genetic testing is rapidly moving health care delivery in a new, and very promising direction.  Sometimes we can test for disease risk before disease symptoms occur.  University of Washington Medical Center is conducting research on genetic laboratory tests to screen large numbers of regular individuals to find people at high risk of developing common diseases such as high cholesterol, iron overload, breast cancer, and colon cancer.  Research genetic screening results will be returned to participants but will not be placed into the participant's medical record.  Those participants found to be at higher risk will be asked to follow up with their doctors about prevention and early screening to improve their health outcome.  If you are interested in learning more and to participate in this study, please click this link: [survey-link].  If the link above does not work, try copying and pasting the link address below onto your web browser:  [survey-url]  The above link is unique to you and should not be forwarded to others.  In the course of the survey, you will be asked if you would want to join this research and have your DNA tested.  Thank you for your time and consideration of this research.  Sincerely,  Brian Shirts, MD, PhD  Associate Professor of Laboratory Medicine  University of Washington Medical Center  Brotman Baty Institute for Precision Medicine  [Contact address]  [Contact email]  [Contact phone number] |
| --- |

## **Study information and frequently asked questions provided by the population genetic screening study at UWM**

| **Study Information** |
| --- |
|  |
| **Q. What do I have to do to join the study?** |
| **A.** First, you have no obligation to join this or any research study. There are no negative consequences if you do not want to join. For any questions you can call Dr. Brian Shirts and the study team at [study phone] or e-mail [study email]. |
|  |
| We must collect your DNA for testing. In a week or two, a saliva collection kit with instructions and consent forms will be sent to you via mail. Please read the consent forms carefully. Return the saliva kit and signed consent forms in the return, postage paid, envelope provided. We get less than a teaspoon of saliva samples. **We cannot receive the saliva sample or test your DNA without the signed consent forms.** |
|  |
| To learn more about this research study, please read the following information. You will be asked a few questions in the next few screens. |
|  |
| **Q. What information do you need?** |
| **A.** We need to know your address and phone number to send you a saliva DNA collection kit and consent form. |
|  |
| Once we receive your sample and consent form in the mail, we will ask you to fill out more questions to tell us about your feelings around genetic testing. This survey will take no more than 20 to 30 minutes to complete. You can skip any questions you would rather not answer. |
|  |
|  |
| **Frequently Asked Questions** |
|  |
| **Q. How long will the DNA results take?** |
| **A.** DNA research testing results take a while. It is our goal to get results to all participants within 6 months or sooner. After receiving your results, your participation is complete. However, you are free to contact the Researchers at any time. |
|  |
| **Q. What kind of results will I get?** |
| **A.** Most people will find that they do not have increased risk for inherited disease that can be identified by this screening test. Those people will get an e-mail allowing them to look up their results on a secure study website or can opt to receive a mailed letter with their results. While these results are reassuring, they do not mean that there is no disease risk; it means that there is no increased risk that can be detected by the genetic screening test used in this study. |
|  |
| About one or two percent of people will get a “higher-risk” result. These people will receive a phone call or e-mail from a study Genetic Counselor or the Investigator to discuss the results. This test just measures risk (or chance) of developing a disease. Not everyone with a “higher-risk” result is certain to develop the disease. Because we conduct a screening test that will not go in your medical records, the results should be confirmed with follow-up clinical testing through your doctor. Printable "higher-risk” result reports will be available to you on the secure website so that you can share them with your doctor. |
|  |
| **Q. What about other genetic studies?** |
| **A.** Everyone that participates in this study can also join the University of Washington – Brotman Baty Precision Medicine Institute (BBI) Biorepository and Registry Study. A repository collects and stores samples and data to be used for future precision medicine research. A registry is a list of names of people who don’t mind being contacted occasionally to hear of new research. If you are interested in joining the BBI Repository/Registry study you can indicate this on a separate paper consent form that you will receive with the saliva DNA collection kit. |
|  |
| People who have a “higher-risk” result will also be eligible to join a study to learn more about their genetic risk, and also involve family members who may share their genetic risk. |
|  |
| **Q. Will my information be kept confidential and safe?** |
| **A.** All information you provide us will be confidential and we will make every effort to keep your information and samples safe. |
|  |
| **Q. How long will my samples and information be saved?** |
| **A.** Your samples may be saved indefinitely. |
|  |
| **Q. What if I change my mind?** |
| **A.** You can contact us at any time to remove your information/sample from the study or tell us to not return results to you. |
|  |
| **Q. What are the risks for me joining this study?** |
| **A.** It may be unpleasant to collect a sufficient amount of saliva (spit) for the DNA test. Learning about your genetics can be emotionally stressful. Some people cannot predict how learning genetic information may make them feel until after they have received the results. Talking about private matters and personal feelings may make you feel uncomfortable or embarrassed. Another possible risk is loss of confidentiality or private information should a data breach occur. We will do our best to help you understand your genetic results and to keep your private information secure. |
|  |
| Although The Genetic Information Nondiscrimination Act of 2008 federal law (GINA) has been passed to prevent discrimination based on genetic information, it is possible that taking part in this study might make it harder to gain and/or keep employment or insurance. If you have any questions about your rights as a research participant, you can contact the University of Washington Human Subjects Division at [phone number] or call collect at [phone number]. |
|  |
|  |
| **Q. What are the benefits for me joining this study?** |
| **A.** We cannot predict if you will receive personal benefit from participating in this research. Our hope is that you will gain information on your genetic risks for a screening list of inherited diseases. Your participation may benefit society by helping to advance genetic science. We are hopeful that future generations may benefit from the scientific and medical knowledge we gain, and that better methods will be put in place to start using genetic information to prevent disease. |
|  |
| **Q. Will I get paid for this study?** |
| **A.** No. |
|  |
| **Q. Does this study cost me anything?** |
| **A.** No. The genetic screening test is free. Any follow-up medical care will not be covered by the study. |
|  |
| **Q. Who is funding this research** |
| **A.** This is funded by the Brotman Baty Institute for Precision Medicine at the University of Washington. |
|  |

## **Summary of survey instruments and measures**

|  | **Survey** | | |
| --- | --- | --- | --- |
| **Measures** | T_0_ | T_1_ | T_2_ |
| Basic Demographics | X |  |  |
| Full Demographics |  | X |  |
| Knowledge (adapted from Kaphingst et al., 2012)^9^ |  | X | X |
| Attitudes (adapted from Fraint et al., 2019)^10^ | X |  |  |
| Psychological Impact and Adaptation to Genetic Testing Results |  |  |  |
| Feelings about genomic testing results questionnaire^11^ |  |  | X |
| Psychosocial aspects of hereditary cancer questionnaire (adapted from Eijzenga et al., 2014)^12^ |  |  | X |
| Family Impact and Communication Practices (adapted from Sinicrope at al., 2008)^13^ |  |  | X |
| Intent to share information with relatives | X | X | X |

## **T0 Survey**

| You have answered that you are willing to respond to some questions about your health history and your feelings around genetic testing. Thank you for your interest.   You can skip any questions that you do not want to answer. If you want to continue with DNA testing, the last page will ask for your contact information to send you a DNA collection kit and written consent forms. | | | | |
| --- | --- | --- | --- | --- |
| Have you ever been diagnosed with cancer? | Yes | | | |
|  | No | | | |
| If Yes: | | | | |
| Which type of cancer?  (Check all that apply) | Breast | | | |
|  | Ovarian | | | |
|  | Colon | | | |
|  | Rectal | | | |
|  | Prostate | | | |
|  | Uterine or Endometrial | | | |
|  | Brain | | | |
|  | Lung | | | |
|  | Blood cancer / Leukemia | | | |
|  | Lymphoma | | | |
|  | Kidney | | | |
|  | Bladder | | | |
|  | Stomach | | | |
|  | Bone / Sarcoma | | | |
|  | Pancreatic | | | |
|  | Liver | | | |
|  | Melanoma | | | |
|  | Other Skin Cancer | | | |
| Are you adopted? | Yes | | | |
|  | No | | | |
| Has a close biological relative (mother, father, son, daughter, aunt, uncle) been diagnosed with cancer? | Yes | | | |
|  | No | | | |
|  | Don’t know | | | |
| If Yes: | | | | |
| Which type of cancer?  (Check all that apply) | Breast | | | |
|  | Ovarian | | | |
|  | Colon | | | |
|  | Rectal | | | |
|  | Prostate | | | |
|  | Uterine or Endometrial | | | |
|  | Brain | | | |
|  | Lung | | | |
|  | Blood cancer / Leukemia | | | |
|  | Lymphoma | | | |
|  | Kidney | | | |
|  | Bladder | | | |
|  | Stomach | | | |
|  | Bone / Sarcoma | | | |
|  | Pancreatic | | | |
|  | Liver | | | |
|  | Melanoma | | | |
|  | Other Skin Cancer | | | |
| Have you had a heart attack or coronary artery disease? | Yes | | | |
|  | No | | | |
| Do you have a family history of heart attack or coronary artery disease in your close biological family? | Yes | | | |
|  | No | | | |
|  | Don’t know | | | |
| Please indicate the extent to which you think the following statements would be a factor in your decision to receive or not receive genetic testing for inherited disease risk. | | | | |
|  | Not Important At All | Somewhat Important | Very Important | N/A |
| To learn that I do not have an inherited disease risk. |  |  |  |  |
| To make decisions about having (more) children. |  |  |  |  |
| To alter priorities (personal, career, etc.) if an inherited disease risk is present. |  |  |  |  |
| To be able to mentally prepare myself for what lies ahead if the test reveals that I have inherited disease risk. |  |  |  |  |
| If the test reveals that I have an inherited disease risk, I would want it to be identified early on so that I can prevent or treat the disease. |  |  |  |  |
| I will regret taking the test if an inherited disease risk is identified. |  |  |  |  |
| Participating in genetic testing is against my personal moral code. |  |  |  |  |
| Knowing whether I have an inherited disease risk would not change what I do in life. |  |  |  |  |
| There is no effective cure/treatment for most genetic disease. |  |  |  |  |
| I need to know more about the test. |  |  |  |  |
| I want to know the test is accurate enough. |  |  |  |  |
| My family thinks that I should or should not have genetic testing. |  |  |  |  |
| I want to know if my healthcare provider thinks that I should or should not have genetic testing. |  |  |  |  |
| I am worried that the results will not remain confidential. |  |  |  |  |
| I am worried about losing my health insurance. |  |  |  |  |
| I am afraid that I would lose my job or be discriminated by future employers. |  |  |  |  |
| What people in your life influence your decision making?  (Choose all that apply) | Spouse or Partner | | | |
|  | Parents | | | |
|  | Children | | | |
|  | Grandparents or Grandchildren | | | |
|  | Sisters or Brothers | | | |
|  | Aunts, Uncles, or Cousins | | | |
|  | Friends | | | |
|  | Spiritual leader (Rabbi, Priest, Minister, etc.) | | | |
|  | Coworkers | | | |
|  | Employer | | | |
| If you had genetic testing would you share the results of your test with others? | Yes | | | |
|  | No | | | |
|  | Don’t know | | | |
| If Yes or Don’t know: | | | | |
| Whom would you share them with?  (Choose all that apply) | Spouse or Partner | | | |
|  | Parents | | | |
|  | Children | | | |
|  | Grandparents or Grandchildren | | | |
|  | Sisters or Brothers | | | |
|  | Aunts, Uncles, or Cousins | | | |
|  | Friends | | | |
|  | Spiritual leader (Rabbi, Priest, Minister, etc.) | | | |
|  | Coworkers | | | |
|  | Employer | | | |

## **T1 Survey**

| Thank you for your interest in participating in this research study about inherited risk genes.  Please complete this survey to tell us more about you, your thoughts about genetic testing, and your personal and family history that may be related to familial disease. This information is important for understanding familial disease risk and will help us improve genetic screening for other people. This survey will take approximately 10 to 30 minutes depending on the size of your family. Feel free to skip any questions that you would prefer not to answer. | | | | | |
| --- | --- | --- | --- | --- | --- |
| First Name | Open text box | | | | |
| Last Name | Open text box | | | | |
| Date of Birth | Open text box | | | | |
| Race  (Check all that apply) | White | | | | |
|  | Black / African American | | | | |
|  | American Indian | | | | |
|  | Alaska Native | | | | |
|  | Native Hawaiian | | | | |
|  | Other Pacific Islander | | | | |
|  | Asian | | | | |
|  | Other race | | | | |
|  | Prefer not to answer | | | | |
|  | Don’t know | | | | |
| Hispanic | Yes | | | | |
|  | No | | | | |
| Is there any other way that you would describe yourself in terms of race/ethnicity? | Open text box | | | | |
| Gender | Male | | | | |
|  | Female | | | | |
|  | Other | | | | |
|  | Prefer not to answer | | | | |
| Sex assigned at birth | Male | | | | |
|  | Female | | | | |
|  | Other | | | | |
|  | Prefer not to answer | | | | |
| Sexual Orientation  (Check all that apply) | Asexual | | | | |
|  | Bisexual | | | | |
|  | Lesbian or Gay | | | | |
|  | Queer | | | | |
|  | Straight (not Lesbian or Gay) | | | | |
|  | Something Else | | | | |
|  | Don’t know | | | | |
|  | Prefer not to answer | | | | |
| Education Level | Less than high school | | | | |
|  | High school/GED | | | | |
|  | Some college | | | | |
|  | College graduate | | | | |
|  | Advanced degree | | | | |
| Household Income | Less than $50,000 | | | | |
|  | Greater than $50,000 but less than or equal to $100,000 | | | | |
|  | Greater than $100,000 | | | | |
|  | Prefer not to answer | | | | |
| Living Situation  (Check all that apply) | I live alone | | | | |
|  | I live with my spouse/partner | | | | |
|  | I live with my parents | | | | |
|  | I live with my children | | | | |
|  | Other | | | | |
|  | Prefer not to answer | | | | |
| If Other: | | | | | |
| Other Living Situation | Open text box | | | | |
| This is a short questionnaire to evaluate you for familial heart disease risk. | | | | | |
| Have you ever had a heart attack or coronary artery disease? | Yes | | | | |
|  | No | | | | |
| If Yes: | | | | | |
| How many heart attacks or coronary artery interventions? (If more than 5, please enter 5) | [The next set of statements populated according to the number stated by the participant on this field] | | | | |
| First type of heart intervention | Heart attack | | | | |
|  | Heart stent put in | | | | |
|  | Stroke | | | | |
|  | Other | | | | |
| If Other: | | | | | |
| Other first type of heart intervention (please describe) | Open text box | | | | |
| Age of first intervention | Open text box | | | | |
| Second type of heart intervention | Heart attack | | | | |
|  | Heart stent put in | | | | |
|  | Stroke | | | | |
|  | Other | | | | |
| If Other: | | | | | |
| Other second type of heart intervention (please describe) | Open text box | | | | |
| Age of second intervention | Open text box | | | | |
| Third type of heart intervention | Heart attack | | | | |
|  | Heart stent put in | | | | |
|  | Stroke | | | | |
|  | Other | | | | |
| If Other: | | | | | |
| Other third type of heart intervention (please describe) | Open text box | | | | |
| Age of third intervention | Open text box | | | | |
| Fourth type of heart intervention | Heart attack | | | | |
|  | Heart stent put in | | | | |
|  | Stroke | | | | |
|  | Other | | | | |
| If Other: | | | | | |
| Other fourth type of heart intervention (please describe) | Open text box | | | | |
| Age of fourth intervention | Open text box | | | | |
| Fifth type of heart intervention | Heart attack | | | | |
|  | Heart stent put in | | | | |
|  | Stroke | | | | |
|  | Other | | | | |
| If Other: | | | | | |
| Other fifth type of heart intervention (please describe) | Open text box | | | | |
| Age of fifth intervention | Open text box | | | | |
| Has your doctor ever prescribed you a medication for lipid levels? | Yes | | | | |
|  | No | | | | |
|  | Don’t know | | | | |
| If Yes: | | | | | |
| How old were you when you were first prescribed this medication? | Open text box | | | | |
| This is a questionnaire to evaluate your biological family for familial heart disease risk. | | | | | |
| Have any of your biological family members ever had heart attacks and/or coronary artery disease?  (Check all that apply) | Mother | | | | |
|  | Father | | | | |
|  | Brother | | | | |
|  | Sister | | | | |
|  | Child | | | | |
|  | None | | | | |
| If Mother: | | | | | |
| Age at diagnosis of mother | Open text box | | | | |
| If Father: | | | | | |
| Age at diagnosis of father | Open text box | | | | |
| If Brother: | | | | | |
| How many brothers have had a heart attack or coronary artery disease?  (If you have more than 3 brothers with heart disease, please enter 3 and information about the brothers with heart disease at the youngest ages.) | [1 – 3. The next 3 statements populated according to the number stated by the participant on this field] | | | | |
| Age at diagnosis of first brother | Open text box | | | | |
| Age at diagnosis of second brother | Open text box | | | | |
| Age at diagnosis of third brother | Open text box | | | | |
| If Sister: | | | | | |
| How many sisters have had a heart attack or coronary artery disease?  (If you have more than 3 sisters with heart disease, please enter 3 and information about the sisters with heart disease at the youngest ages.) | [1 – 3. The next 3 statements populated according to the number stated by the participant on this field] | | | | |
| Age at diagnosis of first sister | Open text box | | | | |
| Age at diagnosis of second sister | Open text box | | | | |
| Age at diagnosis of third sister | Open text box | | | | |
| If Children: | | | | | |
| How many children have had a heart attack or coronary artery disease?  (If you have more than 3 children with heart disease, please enter 3 and information about the children with heart disease at the youngest ages.) | [1 – 3. The next 3 statements populated according to the number stated by the participant on this field] | | | | |
| Age at diagnosis of first child | Open text box | | | | |
| Age at diagnosis of second child | Open text box | | | | |
| Age at diagnosis of third child | Open text box | | | | |
| This is a questionnaire to evaluate your biological relatives from each side of your parents for familial heart disease risk. | | | | | |
| On your mother’s side of the family, have any of the following relatives had heart attacks or coronary artery disease?  (Check all that apply) | Aunt | | | | |
|  | Uncle | | | | |
|  | Grandmother | | | | |
|  | Grandfather | | | | |
|  | Cousin | | | | |
|  | None | | | | |
| If Aunt: | | | | | |
| How many aunts on your mother’s side have had a heart attack or coronary artery disease?  (If you have more than 3 aunts with heart disease, please enter 3 and information about the aunts with heart disease at the youngest ages.) | [1 – 3. The next 3 statements populated according to the number stated by the participant on this field] | | | | |
| Age at diagnosis of first aunt | Open text box | | | | |
| Age at diagnosis of second aunt | Open text box | | | | |
| Age at diagnosis of third aunt | Open text box | | | | |
| If Uncle: | | | | | |
| How many uncles on your mother’s side have had a heart attack or coronary artery disease?  (If you have more than 3 uncles with heart disease, please enter 3 and information about the uncles with heart disease at the youngest ages.) | [1 – 3. The next 3 statements populated according to the number stated by the participant on this field] | | | | |
| Age at diagnosis of first uncle | Open text box | | | | |
| Age at diagnosis of second uncle | Open text box | | | | |
| Age at diagnosis of third uncle | Open text box | | | | |
| If Grandmother: | | | | | |
| Age at diagnosis of grandmother | Open text box | | | | |
| If Grandfather: | | | | | |
| Age at diagnosis of grandfather | Open text box | | | | |
| If Cousin: | | | | | |
| How many cousins on your mother's side have had a heart attack or coronary artery disease? (If you have more than 3 cousins with heart disease, please enter 3 and information about the cousins with heart disease at the youngest ages.) | [1 – 3. The next 3 statements populated according to the number stated by the participant on this field] | | | | |
| Age at diagnosis of first cousin | Open text box | | | | |
| Age at diagnosis of second cousin | Open text box | | | | |
| Age at diagnosis of third cousin | Open text box | | | | |
| On your father’s side of the family, have any of the following relatives had heart attacks or coronary artery disease?  (Check all that apply) | Aunt | | | | |
|  | Uncle | | | | |
|  | Grandmother | | | | |
|  | Grandfather | | | | |
|  | Cousin | | | | |
|  | None | | | | |
| If Aunt: | | | | | |
| How many aunts on your father’s side have had a heart attack or coronary artery disease?  (If you have more than 3 aunts with heart disease, please enter 3 and information about the aunts with heart disease at the youngest ages.) | [1 – 3. The next 3 statements populated according to the number stated by the participant on this field] | | | | |
| Age at diagnosis of first aunt | Open text box | | | | |
| Age at diagnosis of second aunt | Open text box | | | | |
| Age at diagnosis of third aunt | Open text box | | | | |
| If Uncle: | | | | | |
| How many uncles on your father’s side have had a heart attack or coronary artery disease?  (If you have more than 3 uncles with heart disease, please enter 3 and information about the uncles with heart disease at the youngest ages.) | [1 – 3. The next 3 statements populated according to the number stated by the participant on this field] | | | | |
| Age at diagnosis of first uncle | Open text box | | | | |
| Age at diagnosis of second uncle | Open text box | | | | |
| Age at diagnosis of third uncle | Open text box | | | | |
| If Grandmother: | | | | | |
| Age at diagnosis of grandmother | Open text box | | | | |
| If Grandfather: | | | | | |
| Age at diagnosis of grandfather | Open text box | | | | |
| If Cousin: | | | | | |
| How many cousins on your father’s side have had a heart attack or coronary artery disease?  (If you have more than 3 cousins with heart disease, please enter 3 and information about the cousins with heart disease at the youngest ages.) | [1 – 3. The next 3 statements populated according to the number stated by the participant on this field] | | | | |
| Age at diagnosis of first cousin | Open text box | | | | |
| Age at diagnosis of second cousin | Open text box | | | | |
| Age at diagnosis of third cousin | Open text box | | | | |
| Do you have any other relatives who had heart attacks or coronary artery disease? | Yes | | | | |
|  | No | | | | |
| If Yes: | | | | | |
| How many of your other relatives have had a heart attack or coronary artery disease?  (If you have more than 3 other relatives with heart disease, please enter 3 and information about the other relatives with heart disease at the youngest ages.) | [1 – 3. The next 3 statements populated according to the number stated by the participant on this field] | | | | |
| Relationship of first relative | Open text box | | | | |
| Age at diagnosis of first relative | Open text box | | | | |
| Relationship of second relative | Open text box | | | | |
| Age at diagnosis of second relative | Open text box | | | | |
| Relationship of third relative | Open text box | | | | |
| Age at diagnosis of third relative | Open text box | | | | |
| This is a short questionnaire to evaluate you for familial cancer risk. | | | | | |
| Have you ever had a bone marrow transplant? | Yes | | | | |
|  | No | | | | |
| Have you ever had cancer? | Yes | | | | |
|  | No | | | | |
| If Yes: | | | | | |
| How many separate, unrelated, cancers? | [1 – 5. The next 5 statements populated according to the number stated by the participant on this field] | | | | |
| What type of cancer was your first diagnosis? | Breast | | | | |
|  | Ovarian | | | | |
|  | Colon | | | | |
|  | Rectal | | | | |
|  | Prostate | | | | |
|  | Uterine or Endometrial | | | | |
|  | Brain | | | | |
|  | Lung | | | | |
|  | Blood cancer / Leukemia | | | | |
|  | Lymphoma | | | | |
|  | Kidney | | | | |
|  | Bladder | | | | |
|  | Stomach | | | | |
|  | Bone / Sarcoma | | | | |
|  | Pancreatic | | | | |
|  | Liver | | | | |
|  | Melanoma | | | | |
|  | Other Skin Cancer | | | | |
|  | Other Cancer | | | | |
| If Other Cancer: | | | | | |
| What was your first other type of cancer? | Open text box | | | | |
| What was your age at diagnosis of your first cancer? | Open text box | | | | |
| What type of cancer was your second diagnosis? | Breast | | | | |
|  | Ovarian | | | | |
|  | Colon | | | | |
|  | Rectal | | | | |
|  | Prostate | | | | |
|  | Uterine or Endometrial | | | | |
|  | Brain | | | | |
|  | Lung | | | | |
|  | Blood cancer / Leukemia | | | | |
|  | Lymphoma | | | | |
|  | Kidney | | | | |
|  | Bladder | | | | |
|  | Stomach | | | | |
|  | Bone / Sarcoma | | | | |
|  | Pancreatic | | | | |
|  | Liver | | | | |
|  | Melanoma | | | | |
|  | Other Skin Cancer | | | | |
|  | Other Cancer | | | | |
| If Other Cancer: | | | | | |
| What was your second other type of cancer? | Open text box | | | | |
| What was your age at diagnosis of your second cancer? | Open text box | | | | |
| What type of cancer was your third diagnosis? | Breast | | | | |
|  | Ovarian | | | | |
|  | Colon | | | | |
|  | Rectal | | | | |
|  | Prostate | | | | |
|  | Uterine or Endometrial | | | | |
|  | Brain | | | | |
|  | Lung | | | | |
|  | Blood cancer / Leukemia | | | | |
|  | Lymphoma | | | | |
|  | Kidney | | | | |
|  | Bladder | | | | |
|  | Stomach | | | | |
|  | Bone / Sarcoma | | | | |
|  | Pancreatic | | | | |
|  | Liver | | | | |
|  | Melanoma | | | | |
|  | Other Skin Cancer | | | | |
|  | Other Cancer | | | | |
| If Other Cancer: | | | | | |
| What was your third other type of cancer? | Open text box | | | | |
| What was your age at diagnosis of your third cancer? | Open text box | | | | |
| What type of cancer was your fourth diagnosis? | Breast | | | | |
|  | Ovarian | | | | |
|  | Colon | | | | |
|  | Rectal | | | | |
|  | Prostate | | | | |
|  | Uterine or Endometrial | | | | |
|  | Brain | | | | |
|  | Lung | | | | |
|  | Blood cancer / Leukemia | | | | |
|  | Lymphoma | | | | |
|  | Kidney | | | | |
|  | Bladder | | | | |
|  | Stomach | | | | |
|  | Bone / Sarcoma | | | | |
|  | Pancreatic | | | | |
|  | Liver | | | | |
|  | Melanoma | | | | |
|  | Other Skin Cancer | | | | |
|  | Other Cancer | | | | |
| If Other Cancer: | | | | | |
| What was your fourth other type of cancer? | Open text box | | | | |
| What was your age at diagnosis of your fourth cancer? | Open text box | | | | |
| What type of cancer was your fifth diagnosis? | Breast | | | | |
|  | Ovarian | | | | |
|  | Colon | | | | |
|  | Rectal | | | | |
|  | Prostate | | | | |
|  | Uterine or Endometrial | | | | |
|  | Brain | | | | |
|  | Lung | | | | |
|  | Blood cancer / Leukemia | | | | |
|  | Lymphoma | | | | |
|  | Kidney | | | | |
|  | Bladder | | | | |
|  | Stomach | | | | |
|  | Bone / Sarcoma | | | | |
|  | Pancreatic | | | | |
|  | Liver | | | | |
|  | Melanoma | | | | |
|  | Other Skin Cancer | | | | |
|  | Other Cancer | | | | |
| If Other Cancer: | | | | | |
| What was your fifth other type of cancer? | Open text box | | | | |
| What was your age at diagnosis of your fifth cancer? | Open text box | | | | |
| Have you ever had colon polyps removed? | Yes | | | | |
|  | No | | | | |
|  | Don’t know | | | | |
| If Yes: | | | | | |
| Total number of polyps removed? | Open text box | | | | |
| This is a questionnaire to evaluate your biological family for familial cancer risk. | | | | | |
| Have any of your biological family members ever had cancer?  (Check all that apply) | Mother | | | | |
|  | Father | | | | |
|  | Brother | | | | |
|  | Sister | | | | |
|  | Child | | | | |
|  | None | | | | |
| If Mother: | | | | | |
| What type of cancer was your mother’s diagnosis? | Breast | | | | |
|  | Ovarian | | | | |
|  | Colon | | | | |
|  | Rectal | | | | |
|  | Prostate | | | | |
|  | Uterine or Endometrial | | | | |
|  | Brain | | | | |
|  | Lung | | | | |
|  | Blood cancer / Leukemia | | | | |
|  | Lymphoma | | | | |
|  | Kidney | | | | |
|  | Bladder | | | | |
|  | Stomach | | | | |
|  | Bone / Sarcoma | | | | |
|  | Pancreatic | | | | |
|  | Liver | | | | |
|  | Melanoma | | | | |
|  | Other Skin Cancer | | | | |
|  | Other Cancer | | | | |
| If Other Cancer: | | | | | |
| What was your mother’s other type of cancer? | Open text box | | | | |
| Age at diagnosis of mother | Open text box | | | | |
| If Father: | | | | | |
| What type of cancer was your father’s diagnosis? | Breast | | | | |
|  | Ovarian | | | | |
|  | Colon | | | | |
|  | Rectal | | | | |
|  | Prostate | | | | |
|  | Uterine or Endometrial | | | | |
|  | Brain | | | | |
|  | Lung | | | | |
|  | Blood cancer / Leukemia | | | | |
|  | Lymphoma | | | | |
|  | Kidney | | | | |
|  | Bladder | | | | |
|  | Stomach | | | | |
|  | Bone / Sarcoma | | | | |
|  | Pancreatic | | | | |
|  | Liver | | | | |
|  | Melanoma | | | | |
|  | Other Skin Cancer | | | | |
|  | Other Cancer | | | | |
| If Other Cancer: | | | | | |
| What was your father’s other type of cancer? | Open text box | | | | |
| Age at diagnosis of father | Open text box | | | | |
| If Brother: | | | | | |
| How many brothers have had cancer?  (If you have more than 3 brothers with cancer, please enter 3 and information about the brothers with cancer at the youngest ages.) | [1 – 3. The next 3 statements populated according to the number stated by the participant on this field] | | | | |
| What type of cancer was your first brother’s diagnosis? | Breast | | | | |
|  | Ovarian | | | | |
|  | Colon | | | | |
|  | Rectal | | | | |
|  | Prostate | | | | |
|  | Uterine or Endometrial | | | | |
|  | Brain | | | | |
|  | Lung | | | | |
|  | Blood cancer / Leukemia | | | | |
|  | Lymphoma | | | | |
|  | Kidney | | | | |
|  | Bladder | | | | |
|  | Stomach | | | | |
|  | Bone / Sarcoma | | | | |
|  | Pancreatic | | | | |
|  | Liver | | | | |
|  | Melanoma | | | | |
|  | Other Skin Cancer | | | | |
|  | Other Cancer | | | | |
| If Other Cancer: | | | | | |
| What was your first brother’s other type of cancer? | Open text box | | | | |
| Age at diagnosis of first brother | Open text box | | | | |
| What type of cancer was your second brother’s diagnosis? | Breast | | | | |
|  | Ovarian | | | | |
|  | Colon | | | | |
|  | Rectal | | | | |
|  | Prostate | | | | |
|  | Uterine or Endometrial | | | | |
|  | Brain | | | | |
|  | Lung | | | | |
|  | Blood cancer / Leukemia | | | | |
|  | Lymphoma | | | | |
|  | Kidney | | | | |
|  | Bladder | | | | |
|  | Stomach | | | | |
|  | Bone / Sarcoma | | | | |
|  | Pancreatic | | | | |
|  | Liver | | | | |
|  | Melanoma | | | | |
|  | Other Skin Cancer | | | | |
|  | Other Cancer | | | | |
| If Other Cancer: | | | | | |
| What was your second brother’s other type of cancer? | Open text box | | | | |
| Age at diagnosis of second brother | Open text box | | | | |
| What type of cancer was your third brother’s diagnosis? | Breast | | | | |
|  | Ovarian | | | | |
|  | Colon | | | | |
|  | Rectal | | | | |
|  | Prostate | | | | |
|  | Uterine or Endometrial | | | | |
|  | Brain | | | | |
|  | Lung | | | | |
|  | Blood cancer / Leukemia | | | | |
|  | Lymphoma | | | | |
|  | Kidney | | | | |
|  | Bladder | | | | |
|  | Stomach | | | | |
|  | Bone / Sarcoma | | | | |
|  | Pancreatic | | | | |
|  | Liver | | | | |
|  | Melanoma | | | | |
|  | Other Skin Cancer | | | | |
|  | Other Cancer | | | | |
| If Other Cancer: | | | | | |
| What was your third brother’s other type of cancer? | Open text box | | | | |
| Age at diagnosis of third brother | Open text box | | | | |
| If Sister: | | | | | |
| How many sisters have had cancer?  (If you have more than 3 sisters with cancer, please enter 3 and information about the sisters with cancer at the youngest ages.) | [1 – 3. The next 3 statements populated according to the number stated by the participant on this field] | | | | |
| What type of cancer was your first sister’s diagnosis? | Breast | | | | |
|  | Ovarian | | | | |
|  | Colon | | | | |
|  | Rectal | | | | |
|  | Prostate | | | | |
|  | Uterine or Endometrial | | | | |
|  | Brain | | | | |
|  | Lung | | | | |
|  | Blood cancer / Leukemia | | | | |
|  | Lymphoma | | | | |
|  | Kidney | | | | |
|  | Bladder | | | | |
|  | Stomach | | | | |
|  | Bone / Sarcoma | | | | |
|  | Pancreatic | | | | |
|  | Liver | | | | |
|  | Melanoma | | | | |
|  | Other Skin Cancer | | | | |
|  | Other Cancer | | | | |
| If Other Cancer: | | | | | |
| What was your first sister’s other type of cancer? | Open text box | | | | |
| Age at diagnosis of first sister | Open text box | | | | |
| What type of cancer was your second sister’s diagnosis? | Breast | | | | |
|  | Ovarian | | | | |
|  | Colon | | | | |
|  | Rectal | | | | |
|  | Prostate | | | | |
|  | Uterine or Endometrial | | | | |
|  | Brain | | | | |
|  | Lung | | | | |
|  | Blood cancer / Leukemia | | | | |
|  | Lymphoma | | | | |
|  | Kidney | | | | |
|  | Bladder | | | | |
|  | Stomach | | | | |
|  | Bone / Sarcoma | | | | |
|  | Pancreatic | | | | |
|  | Liver | | | | |
|  | Melanoma | | | | |
|  | Other Skin Cancer | | | | |
|  | Other Cancer | | | | |
| If Other Cancer: | | | | | |
| What was your second sister’s other type of cancer? | Open text box | | | | |
| Age at diagnosis of second sister | Open text box | | | | |
| What type of cancer was your third sister’s diagnosis? | Breast | | | | |
|  | Ovarian | | | | |
|  | Colon | | | | |
|  | Rectal | | | | |
|  | Prostate | | | | |
|  | Uterine or Endometrial | | | | |
|  | Brain | | | | |
|  | Lung | | | | |
|  | Blood cancer / Leukemia | | | | |
|  | Lymphoma | | | | |
|  | Kidney | | | | |
|  | Bladder | | | | |
|  | Stomach | | | | |
|  | Bone / Sarcoma | | | | |
|  | Pancreatic | | | | |
|  | Liver | | | | |
|  | Melanoma | | | | |
|  | Other Skin Cancer | | | | |
|  | Other Cancer | | | | |
| If Other Cancer: | | | | | |
| What was your third sister’s other type of cancer? | Open text box | | | | |
| Age at diagnosis of third sister | Open text box | | | | |
| If Child: | | | | | |
| How many children have had cancer?  (If you have more than 3 children with cancer, please enter 3 and information about the children with cancer at the youngest ages.) | [1 – 3. The next 3 statements populated according to the number stated by the participant on this field] | | | | |
| What type of cancer was your first child’s diagnosis? | Breast | | | | |
|  | Ovarian | | | | |
|  | Colon | | | | |
|  | Rectal | | | | |
|  | Prostate | | | | |
|  | Uterine or Endometrial | | | | |
|  | Brain | | | | |
|  | Lung | | | | |
|  | Blood cancer / Leukemia | | | | |
|  | Lymphoma | | | | |
|  | Kidney | | | | |
|  | Bladder | | | | |
|  | Stomach | | | | |
|  | Bone / Sarcoma | | | | |
|  | Pancreatic | | | | |
|  | Liver | | | | |
|  | Melanoma | | | | |
|  | Other Skin Cancer | | | | |
|  | Other Cancer | | | | |
| If Other Cancer: | | | | | |
| What was your first child’s other type of cancer? | Open text box | | | | |
| Age at diagnosis of first child | Open text box | | | | |
| What type of cancer was your second child’s diagnosis? | Breast | | | | |
|  | Ovarian | | | | |
|  | Colon | | | | |
|  | Rectal | | | | |
|  | Prostate | | | | |
|  | Uterine or Endometrial | | | | |
|  | Brain | | | | |
|  | Lung | | | | |
|  | Blood cancer / Leukemia | | | | |
|  | Lymphoma | | | | |
|  | Kidney | | | | |
|  | Bladder | | | | |
|  | Stomach | | | | |
|  | Bone / Sarcoma | | | | |
|  | Pancreatic | | | | |
|  | Liver | | | | |
|  | Melanoma | | | | |
|  | Other Skin Cancer | | | | |
|  | Other Cancer | | | | |
| If Other Cancer: | | | | | |
| What was your second child’s other type of cancer? | Open text box | | | | |
| Age at diagnosis of second child | Open text box | | | | |
| What type of cancer was your third child’s diagnosis? | Breast | | | | |
|  | Ovarian | | | | |
|  | Colon | | | | |
|  | Rectal | | | | |
|  | Prostate | | | | |
|  | Uterine or Endometrial | | | | |
|  | Brain | | | | |
|  | Lung | | | | |
|  | Blood cancer / Leukemia | | | | |
|  | Lymphoma | | | | |
|  | Kidney | | | | |
|  | Bladder | | | | |
|  | Stomach | | | | |
|  | Bone / Sarcoma | | | | |
|  | Pancreatic | | | | |
|  | Liver | | | | |
|  | Melanoma | | | | |
|  | Other Skin Cancer | | | | |
|  | Other Cancer | | | | |
| If Other Cancer: | | | | | |
| What was your third child’s other type of cancer? | Open text box | | | | |
| Age at diagnosis of third child | Open text box | | | | |
| This is a questionnaire to evaluate your biological relatives from each side of your parents for familial cancer risk. | | | | | |
| On your mother’s side of the family, have any of the following relatives had cancer? | Aunt | | | | |
|  | Uncle | | | | |
|  | Grandmother | | | | |
|  | Grandfather | | | | |
|  | Cousin | | | | |
|  | None | | | | |
| If Aunt: | | | | | |
| How many aunts on your mother’s side have had cancer?  (If you have more than 3 aunts with cancer, please enter 3 and information about the aunts with cancer at the youngest ages.) | [1 – 3. The next 3 statements populated according to the number stated by the participant on this field] | | | | |
| What type of cancer was your first aunt’s diagnosis? | Breast | | | | |
|  | Ovarian | | | | |
|  | Colon | | | | |
|  | Rectal | | | | |
|  | Prostate | | | | |
|  | Uterine or Endometrial | | | | |
|  | Brain | | | | |
|  | Lung | | | | |
|  | Blood cancer / Leukemia | | | | |
|  | Lymphoma | | | | |
|  | Kidney | | | | |
|  | Bladder | | | | |
|  | Stomach | | | | |
|  | Bone / Sarcoma | | | | |
|  | Pancreatic | | | | |
|  | Liver | | | | |
|  | Melanoma | | | | |
|  | Other Skin Cancer | | | | |
|  | Other Cancer | | | | |
| If Other Cancer: | | | | | |
| What was your first aunt’s other type of cancer? | Open text box | | | | |
| Age at diagnosis of first aunt | Open text box | | | | |
| What type of cancer was your second aunt’s diagnosis? | Breast | | | | |
|  | Ovarian | | | | |
|  | Colon | | | | |
|  | Rectal | | | | |
|  | Prostate | | | | |
|  | Uterine or Endometrial | | | | |
|  | Brain | | | | |
|  | Lung | | | | |
|  | Blood cancer / Leukemia | | | | |
|  | Lymphoma | | | | |
|  | Kidney | | | | |
|  | Bladder | | | | |
|  | Stomach | | | | |
|  | Bone / Sarcoma | | | | |
|  | Pancreatic | | | | |
|  | Liver | | | | |
|  | Melanoma | | | | |
|  | Other Skin Cancer | | | | |
|  | Other Cancer | | | | |
| If Other Cancer: | | | | | |
| What was your second aunt’s other type of cancer? | Open text box | | | | |
| Age at diagnosis of second aunt | Open text box | | | | |
| What type of cancer was your third aunt’s diagnosis? | Breast | | | | |
|  | Ovarian | | | | |
|  | Colon | | | | |
|  | Rectal | | | | |
|  | Prostate | | | | |
|  | Uterine or Endometrial | | | | |
|  | Brain | | | | |
|  | Lung | | | | |
|  | Blood cancer / Leukemia | | | | |
|  | Lymphoma | | | | |
|  | Kidney | | | | |
|  | Bladder | | | | |
|  | Stomach | | | | |
|  | Bone / Sarcoma | | | | |
|  | Pancreatic | | | | |
|  | Liver | | | | |
|  | Melanoma | | | | |
|  | Other Skin Cancer | | | | |
|  | Other Cancer | | | | |
| If Other Cancer: | | | | | |
| What was your third aunt’s other type of cancer? | Open text box | | | | |
| Age at diagnosis of third aunt | Open text box | | | | |
| If Uncle: | | | | | |
| How many uncles on your mother’s side have had cancer?  (If you have more than 3 uncles with cancer, please enter 3 and information about the uncles with cancer at the youngest ages.) | [1 – 3. The next 3 statements populated according to the number stated by the participant on this field] | | | | |
| What type of cancer was your first uncle’s diagnosis? | Breast | | | | |
|  | Ovarian | | | | |
|  | Colon | | | | |
|  | Rectal | | | | |
|  | Prostate | | | | |
|  | Uterine or Endometrial | | | | |
|  | Brain | | | | |
|  | Lung | | | | |
|  | Blood cancer / Leukemia | | | | |
|  | Lymphoma | | | | |
|  | Kidney | | | | |
|  | Bladder | | | | |
|  | Stomach | | | | |
|  | Bone / Sarcoma | | | | |
|  | Pancreatic | | | | |
|  | Liver | | | | |
|  | Melanoma | | | | |
|  | Other Skin Cancer | | | | |
|  | Other Cancer | | | | |
| If Other Cancer: | | | | | |
| What was your first uncle’s other type of cancer? | Open text box | | | | |
| Age at diagnosis of first uncle | Open text box | | | | |
| What type of cancer was your second uncle’s diagnosis? | Breast | | | | |
|  | Ovarian | | | | |
|  | Colon | | | | |
|  | Rectal | | | | |
|  | Prostate | | | | |
|  | Uterine or Endometrial | | | | |
|  | Brain | | | | |
|  | Lung | | | | |
|  | Blood cancer / Leukemia | | | | |
|  | Lymphoma | | | | |
|  | Kidney | | | | |
|  | Bladder | | | | |
|  | Stomach | | | | |
|  | Bone / Sarcoma | | | | |
|  | Pancreatic | | | | |
|  | Stomach | | | | |
|  | Liver | | | | |
|  | Melanoma | | | | |
|  | Other Skin Cancer | | | | |
|  | Other Cancer | | | | |
| If Other Cancer: | | | | | |
| What was your second uncle’s other type of cancer? | Open text box | | | | |
| Age at diagnosis of second uncle | Open text box | | | | |
| What type of cancer was your third uncle’s diagnosis? | Breast | | | | |
|  | Ovarian | | | | |
|  | Colon | | | | |
|  | Rectal | | | | |
|  | Prostate | | | | |
|  | Uterine or Endometrial | | | | |
|  | Brain | | | | |
|  | Lung | | | | |
|  | Blood cancer / Leukemia | | | | |
|  | Lymphoma | | | | |
|  | Kidney | | | | |
|  | Bladder | | | | |
|  | Stomach | | | | |
|  | Bone / Sarcoma | | | | |
|  | Pancreatic | | | | |
|  | Stomach | | | | |
|  | Liver | | | | |
|  | Melanoma | | | | |
|  | Other Skin Cancer | | | | |
|  | Other Cancer | | | | |
| If Other Cancer: | | | | | |
| What was your third uncle’s other type of cancer? | Open text box | | | | |
| Age at diagnosis of third uncle | Open text box | | | | |
| If Grandmother: | | | | | |
| What type of cancer was your grandmother’s diagnosis? | Breast | | | | |
|  | Ovarian | | | | |
|  | Colon | | | | |
|  | Rectal | | | | |
|  | Prostate | | | | |
|  | Uterine or Endometrial | | | | |
|  | Brain | | | | |
|  | Lung | | | | |
|  | Blood cancer / Leukemia | | | | |
|  | Lymphoma | | | | |
|  | Kidney | | | | |
|  | Bladder | | | | |
|  | Stomach | | | | |
|  | Bone / Sarcoma | | | | |
|  | Pancreatic | | | | |
|  | Liver | | | | |
|  | Melanoma | | | | |
|  | Other Skin Cancer | | | | |
|  | Other Cancer | | | | |
| If Other Cancer: | | | | | |
| What was your grandmother’s other type of cancer? | Open text box | | | | |
| Age at diagnosis of grandmother | Open text box | | | | |
| If Grandfather: | | | | | |
| What type of cancer was your grandfather’s diagnosis? | Breast | | | | |
|  | Ovarian | | | | |
|  | Colon | | | | |
|  | Rectal | | | | |
|  | Prostate | | | | |
|  | Uterine or Endometrial | | | | |
|  | Brain | | | | |
|  | Lung | | | | |
|  | Blood cancer / Leukemia | | | | |
|  | Lymphoma | | | | |
|  | Kidney | | | | |
|  | Bladder | | | | |
|  | Stomach | | | | |
|  | Bone / Sarcoma | | | | |
|  | Pancreatic | | | | |
|  | Liver | | | | |
|  | Melanoma | | | | |
|  | Other Skin Cancer | | | | |
|  | Other Cancer | | | | |
| If Other Cancer: | | | | | |
| What was your grandfather’s other type of cancer? | Open text box | | | | |
| Age at diagnosis of grandfather | Open text box | | | | |
| If Cousin: | | | | | |
| How many cousins on your mother’s side have had cancer?  (If you have more than 3 cousins with cancer, please enter 3 and information about the cousins with cancer at the youngest ages.) | [1 – 3. The next 3 statements populated according to the number stated by the participant on this field] | | | | |
| What type of cancer was your first cousin’s diagnosis? | Breast | | | | |
|  | Ovarian | | | | |
|  | Colon | | | | |
|  | Rectal | | | | |
|  | Prostate | | | | |
|  | Uterine or Endometrial | | | | |
|  | Brain | | | | |
|  | Lung | | | | |
|  | Blood cancer / Leukemia | | | | |
|  | Lymphoma | | | | |
|  | Kidney | | | | |
|  | Bladder | | | | |
|  | Stomach | | | | |
|  | Bone / Sarcoma | | | | |
|  | Pancreatic | | | | |
|  | Liver | | | | |
|  | Melanoma | | | | |
|  | Other Skin Cancer | | | | |
|  | Other Cancer | | | | |
| If Other Cancer: | | | | | |
| What was your first cousin’s other type of cancer? | Open text box | | | | |
| Age at diagnosis of first cousin | Open text box | | | | |
| What type of cancer was your second cousin’s diagnosis? | Breast | | | | |
|  | Ovarian | | | | |
|  | Colon | | | | |
|  | Rectal | | | | |
|  | Prostate | | | | |
|  | Uterine or Endometrial | | | | |
|  | Brain | | | | |
|  | Lung | | | | |
|  | Blood cancer / Leukemia | | | | |
|  | Lymphoma | | | | |
|  | Kidney | | | | |
|  | Bladder | | | | |
|  | Stomach | | | | |
|  | Bone / Sarcoma | | | | |
|  | Pancreatic | | | | |
|  | Liver | | | | |
|  | Melanoma | | | | |
|  | Other Skin Cancer | | | | |
|  | Other Cancer | | | | |
| If Other Cancer: | | | | | |
| What was your second cousin’s other type of cancer? | Open text box | | | | |
| Age at diagnosis of second cousin | Open text box | | | | |
| What type of cancer was your third cousin’s diagnosis? | Breast | | | | |
|  | Ovarian | | | | |
|  | Colon | | | | |
|  | Rectal | | | | |
|  | Prostate | | | | |
|  | Uterine or Endometrial | | | | |
|  | Brain | | | | |
|  | Lung | | | | |
|  | Blood cancer / Leukemia | | | | |
|  | Lymphoma | | | | |
|  | Kidney | | | | |
|  | Bladder | | | | |
|  | Stomach | | | | |
|  | Bone / Sarcoma | | | | |
|  | Pancreatic | | | | |
|  | Liver | | | | |
|  | Melanoma | | | | |
|  | Other Skin Cancer | | | | |
|  | Other Cancer | | | | |
| If Other Cancer: | | | | | |
| What was your third cousin’s other type of cancer? | Open text box | | | | |
| Age at diagnosis of third cousin | Open text box | | | | |
| On your father’s side of the family, have any of the following relatives had cancer?  (Check all that apply) | Aunt | | | | |
|  | Uncle | | | | |
|  | Grandmother | | | | |
|  | Grandfather | | | | |
|  | Cousin | | | | |
|  | None | | | | |
| If Aunt: | | | | | |
| How many aunts on your father’s side have had cancer?  (If you have more than 3 aunts with cancer, please enter 3 and information about the aunts with cancer at the youngest ages.) | [1 – 3. The next 3 statements populated according to the number stated by the participant on this field] | | | | |
| What type of cancer was your first aunt’s diagnosis? | Breast | | | | |
|  | Ovarian | | | | |
|  | Colon | | | | |
|  | Rectal | | | | |
|  | Prostate | | | | |
|  | Uterine or Endometrial | | | | |
|  | Brain | | | | |
|  | Lung | | | | |
|  | Blood cancer / Leukemia | | | | |
|  | Lymphoma | | | | |
|  | Kidney | | | | |
|  | Bladder | | | | |
|  | Stomach | | | | |
|  | Bone / Sarcoma | | | | |
|  | Pancreatic | | | | |
|  | Liver | | | | |
|  | Melanoma | | | | |
|  | Other Skin Cancer | | | | |
|  | Other Cancer | | | | |
| If Other Cancer: | | | | | |
| What was your first aunt’s other type of cancer? | Open text box | | | | |
| Age at diagnosis of first aunt | Open text box | | | | |
| What type of cancer was your second aunt’s diagnosis? | Breast | | | | |
|  | Ovarian | | | | |
|  | Colon | | | | |
|  | Rectal | | | | |
|  | Prostate | | | | |
|  | Uterine or Endometrial | | | | |
|  | Brain | | | | |
|  | Lung | | | | |
|  | Blood cancer / Leukemia | | | | |
|  | Lymphoma | | | | |
|  | Kidney | | | | |
|  | Bladder | | | | |
|  | Stomach | | | | |
|  | Bone / Sarcoma | | | | |
|  | Pancreatic | | | | |
|  | Liver | | | | |
|  | Melanoma | | | | |
|  | Other Skin Cancer | | | | |
|  | Other Cancer | | | | |
| If Other Cancer: | | | | | |
| What was your second aunt’s other type of cancer? | Open text box | | | | |
| Age at diagnosis of second aunt | Open text box | | | | |
| What type of cancer was your third aunt’s diagnosis? | Breast | | | | |
|  | Ovarian | | | | |
|  | Colon | | | | |
|  | Rectal | | | | |
|  | Prostate | | | | |
|  | Uterine or Endometrial | | | | |
|  | Brain | | | | |
|  | Lung | | | | |
|  | Blood cancer / Leukemia | | | | |
|  | Lymphoma | | | | |
|  | Kidney | | | | |
|  | Bladder | | | | |
|  | Stomach | | | | |
|  | Bone / Sarcoma | | | | |
|  | Pancreatic | | | | |
|  | Liver | | | | |
|  | Melanoma | | | | |
|  | Other Skin Cancer | | | | |
|  | Other Cancer | | | | |
| If Other Cancer: | | | | | |
| What was your third aunt’s other type of cancer? | Open text box | | | | |
| Age at diagnosis of third aunt | Open text box | | | | |
| If Uncle: | | | | | |
| How many uncles on your father’s side have had cancer?  (If you have more than 3 uncles with cancer, please enter 3 and information about the uncles with cancer at the youngest ages.) | [1 – 3. The next 3 statements populated according to the number stated by the participant on this field] | | | | |
| What type of cancer was your first uncle’s diagnosis? | Breast | | | | |
|  | Ovarian | | | | |
|  | Colon | | | | |
|  | Rectal | | | | |
|  | Prostate | | | | |
|  | Uterine or Endometrial | | | | |
|  | Brain | | | | |
|  | Lung | | | | |
|  | Blood cancer / Leukemia | | | | |
|  | Lymphoma | | | | |
|  | Kidney | | | | |
|  | Bladder | | | | |
|  | Stomach | | | | |
|  | Bone / Sarcoma | | | | |
|  | Pancreatic | | | | |
|  | Liver | | | | |
|  | Melanoma | | | | |
|  | Other Skin Cancer | | | | |
|  | Other Cancer | | | | |
| If Other Cancer: | | | | | |
| What was your first uncle’s other type of cancer? | Open text box | | | | |
| Age at diagnosis of first uncle | Open text box | | | | |
| What type of cancer was your second uncle’s diagnosis? | Breast | | | | |
|  | Ovarian | | | | |
|  | Colon | | | | |
|  | Rectal | | | | |
|  | Prostate | | | | |
|  | Uterine or Endometrial | | | | |
|  | Brain | | | | |
|  | Lung | | | | |
|  | Blood cancer / Leukemia | | | | |
|  | Lymphoma | | | | |
|  | Kidney | | | | |
|  | Bladder | | | | |
|  | Stomach | | | | |
|  | Bone / Sarcoma | | | | |
|  | Pancreatic | | | | |
|  | Liver | | | | |
|  | Melanoma | | | | |
|  | Other Skin Cancer | | | | |
|  | Other Cancer | | | | |
| If Other Cancer: | | | | | |
| What was your second uncle’s other type of cancer? | Open text box | | | | |
| Age at diagnosis of second uncle | Open text box | | | | |
| What type of cancer was your third uncle’s diagnosis? | Breast | | | | |
|  | Ovarian | | | | |
|  | Colon | | | | |
|  | Rectal | | | | |
|  | Prostate | | | | |
|  | Uterine or Endometrial | | | | |
|  | Brain | | | | |
|  | Lung | | | | |
|  | Blood cancer / Leukemia | | | | |
|  | Lymphoma | | | | |
|  | Kidney | | | | |
|  | Bladder | | | | |
|  | Stomach | | | | |
|  | Bone / Sarcoma | | | | |
|  | Pancreatic | | | | |
|  | Liver | | | | |
|  | Melanoma | | | | |
|  | Other Skin Cancer | | | | |
|  | Other Cancer | | | | |
| If Other Cancer: | | | | | |
| What was your third uncle’s other type of cancer? | Open text box | | | | |
| Age at diagnosis of third uncle | Open text box | | | | |
| If Grandmother: | | | | | |
| What type of cancer was your grandmother’s diagnosis? | Breast | | | | |
|  | Ovarian | | | | |
|  | Colon | | | | |
|  | Rectal | | | | |
|  | Prostate | | | | |
|  | Uterine or Endometrial | | | | |
|  | Brain | | | | |
|  | Lung | | | | |
|  | Blood cancer / Leukemia | | | | |
|  | Lymphoma | | | | |
|  | Kidney | | | | |
|  | Bladder | | | | |
|  | Stomach | | | | |
|  | Bone / Sarcoma | | | | |
|  | Pancreatic | | | | |
|  | Liver | | | | |
|  | Melanoma | | | | |
|  | Other Skin Cancer | | | | |
|  | Other Cancer | | | | |
| If Other Cancer: | | | | | |
| What was your grandmother’s other type of cancer? | Open text box | | | | |
| Age at diagnosis of grandmother | Open text box | | | | |
| If Grandfather: | | | | | |
| What type of cancer was your grandfather’s diagnosis? | Breast | | | | |
|  | Ovarian | | | | |
|  | Colon | | | | |
|  | Rectal | | | | |
|  | Prostate | | | | |
|  | Uterine or Endometrial | | | | |
|  | Brain | | | | |
|  | Lung | | | | |
|  | Blood cancer / Leukemia | | | | |
|  | Lymphoma | | | | |
|  | Kidney | | | | |
|  | Bladder | | | | |
|  | Stomach | | | | |
|  | Bone / Sarcoma | | | | |
|  | Pancreatic | | | | |
|  | Liver | | | | |
|  | Melanoma | | | | |
|  | Other Skin Cancer | | | | |
|  | Other Cancer | | | | |
| If Other Cancer: | | | | | |
| What was your grandfather’s other type of cancer? | Open text box | | | | |
| Age at diagnosis of grandfather | Open text box | | | | |
| If Cousin: | | | | | |
| How many cousins on your father’s side have had cancer?  (If you have more than 3 cousins with cancer, please enter 3 and information about the cousins with cancer at the youngest ages.) | [1 – 3. The next 3 statements populated according to the number stated by the participant on this field] | | | | |
| What type of cancer was your first cousin’s diagnosis? | Breast | | | | |
|  | Ovarian | | | | |
|  | Colon | | | | |
|  | Rectal | | | | |
|  | Prostate | | | | |
|  | Uterine or Endometrial | | | | |
|  | Brain | | | | |
|  | Lung | | | | |
|  | Blood cancer / Leukemia | | | | |
|  | Lymphoma | | | | |
|  | Kidney | | | | |
|  | Bladder | | | | |
|  | Stomach | | | | |
|  | Bone / Sarcoma | | | | |
|  | Pancreatic | | | | |
|  | Liver | | | | |
|  | Melanoma | | | | |
|  | Other Skin Cancer | | | | |
|  | Other Cancer | | | | |
| If Other Cancer: | | | | | |
| What was your first cousin’s other type of cancer? | Open text box | | | | |
| Age at diagnosis of first cousin | Open text box | | | | |
| What type of cancer was your second cousin’s diagnosis? | Breast | | | | |
|  | Ovarian | | | | |
|  | Colon | | | | |
|  | Rectal | | | | |
|  | Prostate | | | | |
|  | Uterine or Endometrial | | | | |
|  | Brain | | | | |
|  | Lung | | | | |
|  | Blood cancer / Leukemia | | | | |
|  | Lymphoma | | | | |
|  | Kidney | | | | |
|  | Bladder | | | | |
|  | Stomach | | | | |
|  | Bone / Sarcoma | | | | |
|  | Pancreatic | | | | |
|  | Liver | | | | |
|  | Melanoma | | | | |
|  | Other Skin Cancer | | | | |
|  | Other Cancer | | | | |
| If Other Cancer: | | | | | |
| What was your second cousin’s other type of cancer? | Open text box | | | | |
| Age at diagnosis of second cousin | Open text box | | | | |
| What type of cancer was your third cousin’s diagnosis? | Breast | | | | |
|  | Ovarian | | | | |
|  | Colon | | | | |
|  | Rectal | | | | |
|  | Prostate | | | | |
|  | Uterine or Endometrial | | | | |
|  | Brain | | | | |
|  | Lung | | | | |
|  | Blood cancer / Leukemia | | | | |
|  | Lymphoma | | | | |
|  | Kidney | | | | |
|  | Bladder | | | | |
|  | Stomach | | | | |
|  | Bone / Sarcoma | | | | |
|  | Pancreatic | | | | |
|  | Liver | | | | |
|  | Melanoma | | | | |
|  | Other Skin Cancer | | | | |
|  | Other Cancer | | | | |
| If Other Cancer: | | | | | |
| What was your third cousin’s other type of cancer? | Open text box | | | | |
| Age at diagnosis of third cousin | Open text box | | | | |
| Do you have any other relatives who have had cancer? | Yes | | | | |
|  | No | | | | |
| If Yes: | | | | | |
| How many of these other relatives have had cancer?  (If you have more than 3 other relatives with cancer, please enter 3 and information about the other relatives with cancer at the youngest ages.) | [1 – 3. The next 3 statements populated according to the number stated by the participant on this field] | | | | |
| Relationship of first relative | Open text box | | | | |
| What type of cancer was your first relative’s diagnosis? | Breast | | | | |
|  | Ovarian | | | | |
|  | Colon | | | | |
|  | Rectal | | | | |
|  | Prostate | | | | |
|  | Uterine or Endometrial | | | | |
|  | Brain | | | | |
|  | Lung | | | | |
|  | Blood cancer / Leukemia | | | | |
|  | Lymphoma | | | | |
|  | Kidney | | | | |
|  | Bladder | | | | |
|  | Stomach | | | | |
|  | Bone / Sarcoma | | | | |
|  | Pancreatic | | | | |
|  | Liver | | | | |
|  | Melanoma | | | | |
|  | Other Skin Cancer | | | | |
|  | Other Cancer | | | | |
| If Other Cancer: | | | | | |
| What was your first relative's other type of cancer? | Open text box | | | | |
| Age at diagnosis of first relative | Open text box | | | | |
| Relationship of second relative | Open text box | | | | |
| What type of cancer was your second relative’s diagnosis? | Breast | | | | |
|  | Ovarian | | | | |
|  | Colon | | | | |
|  | Rectal | | | | |
|  | Prostate | | | | |
|  | Uterine or Endometrial | | | | |
|  | Brain | | | | |
|  | Lung | | | | |
|  | Blood cancer / Leukemia | | | | |
|  | Lymphoma | | | | |
|  | Kidney | | | | |
|  | Bladder | | | | |
|  | Stomach | | | | |
|  | Bone / Sarcoma | | | | |
|  | Pancreatic | | | | |
|  | Liver | | | | |
|  | Melanoma | | | | |
|  | Other Skin Cancer | | | | |
|  | Other Cancer | | | | |
| If Other Cancer: | | | | | |
| What was your second relative's other type of cancer? | Open text box | | | | |
| Age at diagnosis of second relative | Open text box | | | | |
| Relationship of third relative | Open text box | | | | |
| What type of cancer was your third relative’s diagnosis? | Breast | | | | |
|  | Ovarian | | | | |
|  | Colon | | | | |
|  | Rectal | | | | |
|  | Prostate | | | | |
|  | Uterine or Endometrial | | | | |
|  | Brain | | | | |
|  | Lung | | | | |
|  | Blood cancer / Leukemia | | | | |
|  | Lymphoma | | | | |
|  | Kidney | | | | |
|  | Bladder | | | | |
|  | Stomach | | | | |
|  | Bone / Sarcoma | | | | |
|  | Pancreatic | | | | |
|  | Liver | | | | |
|  | Melanoma | | | | |
|  | Other Skin Cancer | | | | |
|  | Other Cancer | | | | |
| If Other Cancer: | | | | | |
| What was your third relative's other type of cancer? | Open text box | | | | |
| Age at diagnosis of third relative | Open text box | | | | |
| Have you had genetic testing in the past? | Yes | | | | |
|  | No | | | | |
| If Yes: | | | | | |
| What type of genetic testing have you had? | Open text box | | | | |
| How many years ago did you have genetic testing? | Open text box | | | | |
| Has anyone in your family had a genetic test that showed increased disease risk? | Yes | | | | |
|  | No | | | | |
| If Yes: | | | | | |
| What is their relationship to you? (You may enter more than one relative, if several have had genetic testing) | Open text box | | | | |
| What is their genetic test result (if known)? | Open text box | | | | |
| Please indicate how strongly you agree or disagree with the following statements. | | | | | |
|  | STRONGLY DISAGREE (1) | (2) | (3) | (4) | STRONGLY AGREE (5) |
| Once a variant in a gene that affects a person's risk of a disease is found, that disease can always be prevented or cured. |  |  |  |  |  |
| A health care provider can tell a person their exact chances of developing a disease based on the results from genetic testing. |  |  |  |  |  |
| Scientists know how all variants of genes will affect a person's chance of developing diseases. |  |  |  |  |  |
| Even if a person has a variant in a gene that affects their risk of a disease, they may not develop that disease. |  |  |  |  |  |
| Most people can't get genetic testing through their physician's office. |  |  |  |  |  |
| Genetic testing may find variants in a person's genes that can then be passed on to their children. |  |  |  |  |  |
| Genetic testing available today may give a person information about their chances of developing several different diseases. |  |  |  |  |  |
| Genetic testing available today may find variants in a person's genes that will increase their chances of developing a disease in their lifetime. |  |  |  |  |  |
| Genetic testing available today may find variants in a person's genes that will decrease their chances of developing a disease in their lifetime. |  |  |  |  |  |
| Genetic testing available today may find variants in a person's genes that may determine how they respond to certain medications. |  |  |  |  |  |
| A person's health habits, like diet and exercise, can affect whether or not their genes cause diseases. |  |  |  |  |  |
| Do you think you would be willing to share the results of your genetic test with relatives or friends? | Yes | | | | |
|  | No | | | | |
|  | Don’t know | | | | |
| If Yes or Don’t know: | | | | | |
| With whom would you share your genetic results with?  (Check all that apply) | Spouse | | | | |
|  | Parents | | | | |
|  | Children | | | | |
|  | Grandparents / Grandchildren | | | | |
|  | Brothers / Sisters | | | | |
|  | Aunts / Uncles / Cousins | | | | |
|  | Friends | | | | |
|  | Spiritual Leader (Rabbi, Priest, Minister, etc.) | | | | |
|  | Coworkers | | | | |
|  | Employer | | | | |
| Will you please tell us more about why you would or would not share your genetic test results with the people you indicated above? | Open text box | | | | |

## **T2 Survey**

| The following questions ask about your medical plans after receiving your genetic test results… | | | | | | | | | | | | | | | | | | | | |
| --- | --- | --- | --- | --- | --- | --- | --- | --- | --- | --- | --- | --- | --- | --- | --- | --- | --- | --- | --- | --- |
| Do you think your risk of cancer has changed now that you have the result of your test? | | | | | | | Yes | | | | | | | | | | | | | |
|  |  |  |  |  |  |  | No | | | | | | | | | | | | | |
| If Yes: | | | | | | | | | | | | | | | | | | | | |
| Do you think you have higher or lower cancer risk? | | | | | | | Higher | | | | | | | | | | | | | |
|  |  |  |  |  |  |  | Lower | | | | | | | | | | | | | |
| Could you let us know why you think your risk has changed? | | | | | | | Open text box | | | | | | | | | | | | | |
| Do you think your risk of heart disease has changed now that you have the result of your test? | | | | | | | Yes | | | | | | | | | | | | | |
|  |  |  |  |  |  |  | No | | | | | | | | | | | | | |
| If Yes: | | | | | | | | | | | | | | | | | | | | |
| Do you think you have higher or lower heart disease risk? | | | | | | | Higher | | | | | | | | | | | | | |
|  |  |  |  |  |  |  | Lower | | | | | | | | | | | | | |
| Could you let us know why you think your risk has changed? | | | | | | | Open text box | | | | | | | | | | | | | |
| Do you plan on making changes to your health care because of your genetic test results? | | | | | | | Yes | | | | | | | | | | | | | |
|  |  |  |  |  |  |  | No | | | | | | | | | | | | | |
| If Yes: | | | | | | | | | | | | | | | | | | | | |
| How will your health care change because of your genetic test results? | | | | | | | Open text box | | | | | | | | | | | | | |
| Do you plan on doing anything else differently based on your genetic test results? | | | | | | | Yes | | | | | | | | | | | | | |
|  |  |  |  |  |  |  | No | | | | | | | | | | | | | |
| If Yes: | | | | | | | | | | | | | | | | | | | | |
| Please describe how or what you would do differently based on your genetic test result: | | | | | | | Open text box | | | | | | | | | | | | | |
| Have you or do you plan to share your genetic results with your doctor? | | | | | | | Yes | | | | | | | | | | | | | |
|  |  |  |  |  |  |  | No | | | | | | | | | | | | | |
|  |  |  |  |  |  |  | Don’t know | | | | | | | | | | | | | |
| Have you or do you plan to share the results of your genetic test with relatives or friends? | | | | | | | Yes | | | | | | | | | | | | | |
|  |  |  |  |  |  |  | No | | | | | | | | | | | | | |
|  |  |  |  |  |  |  | Don’t know | | | | | | | | | | | | | |
| If Yes or Don’t know: | | | | | | | | | | | | | | | | | | | | |
| With whom do you plan to share the results of your genetic test? (Check all that apply) | | | | | | | Spouse | | | | | | | | | | | | | |
|  |  |  |  |  |  |  | Parents | | | | | | | | | | | | | |
|  |  |  |  |  |  |  | Children | | | | | | | | | | | | | |
|  |  |  |  |  |  |  | Grandparents / Grandchildren | | | | | | | | | | | | | |
|  |  |  |  |  |  |  | Brothers / Sisters | | | | | | | | | | | | | |
|  |  |  |  |  |  |  | Aunts / Uncles / Cousins | | | | | | | | | | | | | |
|  |  |  |  |  |  |  | Friends | | | | | | | | | | | | | |
|  |  |  |  |  |  |  | Spiritual Leader (Rabbi, Priest, Minister, etc.) | | | | | | | | | | | | | |
|  |  |  |  |  |  |  | Coworkers | | | | | | | | | | | | | |
|  |  |  |  |  |  |  | Employer | | | | | | | | | | | | | |
| Will you please tell us more about why you plan to share your genetic test results with the people you indicated above? | | | | | | | Open text box | | | | | | | | | | | | | |
| The following questions ask about how you felt after receiving your genetic test results. Please indicate how deeply you felt in the past week… | | | | | | | | | | | | | | | | | | | | |
|  | | NOT AT ALL | | | A LITTLE | | | | SOMEWHAT | | | | A GOOD DEAL | | | | A GREAT DEAL | | | |
| How upset did you feel about your genetic test result? | |  | | |  | | | |  | | | |  | | | |  | | | |
| How happy did you feel about your genetic test result? | |  | | |  | | | |  | | | |  | | | |  | | | |
| How anxious or nervous did you feel about your genetic test result? | |  | | |  | | | |  | | | |  | | | |  | | | |
| How relieved did you feel about your genetic test result? | |  | | |  | | | |  | | | |  | | | |  | | | |
| How sad did you feel about your genetic test result? | |  | | |  | | | |  | | | |  | | | |  | | | |
| How frustrated did you feel that there are no definite disease prevention guidelines for you? | |  | | |  | | | |  | | | |  | | | |  | | | |
| How uncertain did you feel about what your genetic test result means for you? | |  | | |  | | | |  | | | |  | | | |  | | | |
| How uncertain did you feel about what your genetic test result means for your child(ren) and/or family's risk of disease? | |  | | |  | | | |  | | | |  | | | |  | | | |
| How much did you feel that you clearly understood your choices for disease prevention or early detection? | |  | | |  | | | |  | | | |  | | | |  | | | |
| How concerned did you feel that your genetic test result would affect your health insurance status? | |  | | |  | | | |  | | | |  | | | |  | | | |
| How helpful was the information you received from your genetic test result in planning for the future? | |  | | |  | | | |  | | | |  | | | |  | | | |
| How concerned did you feel that your genetic test result would affect your employment status? | |  | | |  | | | |  | | | |  | | | |  | | | |
| Please rate your feelings towards your hereditary predisposition in regards to genetic testing. | | | | | | | | | | | | | | | | | | | | |
|  | NOT AT ALL | | | A LITTLE | | SOMEWHAT | | | | | | A GOOD DEAL | | | | A GREAT DEAL | | | | N/A |
| Are you worried about the chance of being a carrier of a genetic mutation? |  | | |  | |  | | | | | |  | | | |  | | | |  |
| Are you worried about having to choose whether or not to go for genetic counseling and testing? |  | | |  | |  | | | | | |  | | | |  | | | |  |
| Are you worried about the choice of possible preventative options (screening or surgery)? |  | | |  | |  | | | | | |  | | | |  | | | |  |
| Are you worried about coping with the (future) DNA test results? |  | | |  | |  | | | | | |  | | | |  | | | |  |
| Are you worried about (fulfilling) your plans for having children? |  | | |  | |  | | | | | |  | | | |  | | | |  |
| Please rate your feelings towards your family and your social environment in regards to genetic testing. | | | | | | | | | | | | | | | | | | | | |
|  | NOT AT ALL | | | A LITTLE | | SOMEWHAT | | | | | | A GOOD DEAL | | | | A GREAT DEAL | | | | N/A |
| Do you feel misunderstood by your partner/family/social circle with respect to genetic testing? |  | | |  | |  | | | | | |  | | | |  | | | |  |
| Are you bothered by the lack of support about genetic testing from your partner/family/social circle? |  | | |  | |  | | | | | |  | | | |  | | | |  |
| Are you worried about your immediate family's functionality because of genetic testing? |  | | |  | |  | | | | | |  | | | |  | | | |  |
| Are you worried about contacting family members about genetic testing? |  | | |  | |  | | | | | |  | | | |  | | | |  |
| Are you worried about coping with cancer within the family? |  | | |  | |  | | | | | |  | | | |  | | | |  |
| Are you burdened by feelings of responsibility towards family members related to genetic testing? |  | | |  | |  | | | | | |  | | | |  | | | |  |
| Living with Cancer | | | | | | | | | | | | | | | | | | | | |
| Have you or your family members ever had cancer? | | | Yes | | | | | | | | | | | | | | | | | |
|  |  |  | No | | | | | | | | | | | | | | | | | |
| If Yes: | | | | | | | | | | | | | | | | | | | | |
|  | NOT AT ALL | | | A LITTLE | | SOMEWHAT | | | | | | A GOOD DEAL | | | | A GREAT DEAL | | | | N/A |
| How emotionally burdensome is it for you that you or your family member(s) had cancer? |  | | |  | |  | | | | | |  | | | |  | | | |  |
| How emotionally burdensome is it or would it be to lose a family member because of cancer? |  | | |  | |  | | | | | |  | | | |  | | | |  |
| How emotionally burdensome is your or your family member's diagnosis or treatment of cancer? |  | | |  | |  | | | | | |  | | | |  | | | |  |
| Are you worried about the chance of you getting cancer (again)? |  | | |  | |  | | | | | |  | | | |  | | | |  |
| Are you worried about the chance that your family member(s) will get cancer? |  | | |  | |  | | | | | |  | | | |  | | | |  |
| Living with Heart Disease | | | | | | | | | | | | | | | | | | | | |
| Have you or your family members ever had heart disease? | | | Yes | | | | | | | | | | | | | | | | | |
|  |  |  | No | | | | | | | | | | | | | | | | | |
| If Yes: | | | | | | | | | | | | | | | | | | | | |
|  | NOT AT ALL | | | A LITTLE | | SOMEWHAT | | | | | | A GOOD DEAL | | | | A GREAT DEAL | | | | N/A |
| How emotionally burdensome is it for you that you or your family member(s) have heart disease? |  | | |  | |  | | | | | |  | | | |  | | | |  |
| How emotionally burdensome is it or would it be to lose a family member because of heart disease? |  | | |  | |  | | | | | |  | | | |  | | | |  |
| How emotionally burdensome is your or your family member's diagnosis or treatment for heart disease? |  | | |  | |  | | | | | |  | | | |  | | | |  |
| Are you worried about the chance of you getting heart disease? |  | | |  | |  | | | | | |  | | | |  | | | |  |
| Are you worried about the chance that your family member(s) will get heart disease? |  | | |  | |  | | | | | |  | | | |  | | | |  |
| Do you have children? | | | Yes | | | | | | | | | | | | | | | | | |
|  |  |  | No | | | | | | | | | | | | | | | | | |
| If Yes: | | | | | | | | | | | | | | | | | | | | |
|  | NOT AT ALL | | | A LITTLE | | | | | | SOMEWHAT | | | | A GOOD DEAL | | | | A GREAT DEAL | | |
| Do you feel guilty about the chance of passing on to your children your possible genetic alterations? |  | | |  | | | | | |  | | | |  | | | |  | | |
| Are you worried about telling your children the results? |  | | |  | | | | | |  | | | |  | | | |  | | |
| Are you worried about the chance of your children developing cancer and/or heart disease? |  | | |  | | | | | |  | | | |  | | | |  | | |
| Are there any other issues related to genetic testing that bother you or that you are worried about? | | | Yes | | | | | | | | | | | | | | | | | |
|  |  |  | No | | | | | | | | | | | | | | | | | |
| If Yes: | | | | | | | | | | | | | | | | | | | | |
| What other issues bother or worry you that are related to genetic testing? | | | Open text box | | | | | | | | | | | | | | | | | |
| Do you have a family history of cancer? | | | Yes | | | | | | | | | | | | | | | | | |
|  |  |  | No | | | | | | | | | | | | | | | | | |
| If Yes, questions in red below will also populate: | | |  | | | | | | | | | | | | | | | | | |
|  | | | NOT AT ALL | | | | | A LITTLE | | | SOMEWHAT | | | | A GOOD DEAL | | | | A GREAT DEAL | |
| There are one or two people in my family who usually keep us informed about cancer. | | |  | | | | |  | | |  | | | |  | | | |  | |
| I have learned most of what I know about cancer from my family. | | |  | | | | |  | | |  | | | |  | | | |  | |
| I think other people pity our family because we have so many people with cancer. | | |  | | | | |  | | |  | | | |  | | | |  | |
| Our family is not really very different from most families. | | |  | | | | |  | | |  | | | |  | | | |  | |
| Because of having cancer in our family, some other people think our family is not a normal family. | | |  | | | | |  | | |  | | | |  | | | |  | |
| My family sees cancer as a curse on the family. | | |  | | | | |  | | |  | | | |  | | | |  | |
| Having cancer in our family has torn us apart in some ways. | | |  | | | | |  | | |  | | | |  | | | |  | |
| In my family, cancer is seen as a family issue rather than just a problem for the people who have cancer. | | |  | | | | |  | | |  | | | |  | | | |  | |
| Having cancer in my family has made us a close (closer) family. | | |  | | | | |  | | |  | | | |  | | | |  | |
| People in my family who have had cancer seem to be united by a special bond | | |  | | | | |  | | |  | | | |  | | | |  | |
| Most of my family is uncomfortable about discussing cancer. | | |  | | | | |  | | |  | | | |  | | | |  | |
| Cancer is an open topic in my family. | | |  | | | | |  | | |  | | | |  | | | |  | |
| My family usually tries to keep the children from hearing conversations about cancer. | | |  | | | | |  | | |  | | | |  | | | |  | |
| For the most part, people in my family who have had cancer only talk to each other about it. | | |  | | | | |  | | |  | | | |  | | | |  | |
| People in my family who have had cancer generally keep their feelings about it to themselves. | | |  | | | | |  | | |  | | | |  | | | |  | |
| I feel a lot of pressure from my family to do things to avoid getting cancer. | | |  | | | | |  | | |  | | | |  | | | |  | |
| Some people in my family are critical of the relatives who don't take care of their health. | | |  | | | | |  | | |  | | | |  | | | |  | |
| Someone in my family often reminds me to do things to avoid getting cancer. | | |  | | | | |  | | |  | | | |  | | | |  | |

## **Example uninformative results letter**

**Research Genetic Screening Report**

**Results:** No actionable DNA changes detected in the genes tested.

**Interpretation:** We checked your DNA in the genes listed below for some of the changes that are known to cause familial cancer and cardiovascular disease. We did not find any changes that are known to cause disease risk. This is a screening test, not a diagnostic test, so there could be changes in your DNA that we could not detect. This result suggests that your risk of cancer and cardiovascular disease is most likely to be the same as the risk of other people in the general population with the same age and lifestyle.

**Common Questions:**

**Q.**  Does this mean that I do not have genetic risk of cancer or cardiovascular disease?

**A.** No. We did not detect cancer and cardiovascular disease risk at the genes tested, but there are other genetic and non-genetic causes of disease risk. If you have more questions about your individual disease risk you should talk to your doctor.

**Q.** Was this a full genome test?

**A.**  No. We just looked at parts of a few genes for cancer and heart disease risk.

**Q.** How were these genes chosen?

**A.** These genes are some of the genes most commonly tested in people with familial cancer and familial cardiovascular disease. If an actionable genetic variant is found in these genes, people can take specific actions to prevent disease.

**Q.** Should I show this result to my doctor?

**A.** You may share this result with your doctor, but there is no action that your doctor should take because of this test result. Your doctor can tell you about the disease prevention plan that is best for you based on your lifestyle, family history, and personal preferences.

*If you have other questions about this result you can contact study medical staff at* [study phone] *or* [study email]

**Genes tested:** APC, ATM, BMPR1A, BRCA1, BRCA2, BRIP1, CDH1, CHEK2, EPCAM, HOXB13, LDLR, MLH1, MSH2, MSH6, MUTYH, NTHL1, PALB2, PMS2, PTEN, RAD51C, RAD51D, SMAD4, STK11, TP53

**Limitations:** This test may not detect all variants in the genes analyzed. It is designed to be a screening test, not a diagnostic test. This test was done on DNA from saliva collected by research participants. Sample labels were not checked by a health care provider at time of sample collection. Saliva DNA comes mostly from white blood cells. For some people, such as people who have received a bone marrow transplant, this test will not accurately assess inherited risk. If you are concerned that this test may have missed something, you should talk to your doctor about diagnostic genetic testing.

## **Example positive results letter**

**Research Genetic Screening Report**

**Results:** Actionable DNA change detected in the BRCA2 gene.

**Interpretation:** We detected a change in the BRCA2 gene that is thought to cause increased risk for breast cancer, ovarian cancer, and other cancers. If this result is confirmed with diagnostic testing, it would mean that you are more likely to get certain types of cancer than other people. This result does not mean you have cancer. This result does not mean that you are certain to get cancer. You should have already received a call from a study genetic counselor to discuss these results with you. If you have not, please call [study phone] to learn more about these results and next steps. Because of this screening result, you should talk to your doctor about getting a confirmatory genetic test that includes the *BRCA2* gene. Your doctor is welcome to contact us at [study phone] or [study email] for more details about this result. If this result is confirmed, your doctor may suggest things you can do to prevent cancer.

We checked your DNA for other genes listed below for some of the changes that are known to cause familial cancer and cardiovascular disease. We did not find any changes that are known to cause disease risk in the other genes listed below. This is a screening test, not a diagnostic test, so there could be changes in your DNA that we could not detect.

**Common Questions:**

**Q.** Should I show this result to my doctor?

**A. *Yes!*** You should share this result with your doctor. You doctor can order a diagnostic test to confirm this screening result. If confirmed your doctor can use this information to create a disease prevention plan that is best for you based on your genetic results, lifestyle, family history, and personal preferences.

**Q.** Will others in my family have the same BRCA2 gene change?

**A.** Genetic changes are usually inherited, so it is likely that one of your parents and half of your brothers and sisters will have the same genetic change. There can be new genetic changes that are not inherited, but this is not common. If you have children, they may have inherited the same genetic change. Others in your family may benefit from knowing about your result. It is important for them to talk to their doctors before making any changes in their medications or their medical treatment.

**Q.** Was this a full genome test?

**A.**  No. We just looked at parts of a few genes for cancer and heart disease risk.

**Q.** How were these genes chosen?

**A.** These genes are some of the genes most commonly tested in people with familial cancer and familial cardiovascular disease. If an actionable genetic variant is found in these genes, people can take specific actions to prevent disease.

*If you have other questions about this result you can contact study medical staff at* [study phone] *or* [study email]

**Genes tested:** APC, ATM, BMPR1A, BRCA1, BRCA2, BRIP1, CDH1, CHEK2, EPCAM, HFE, HOXB13, LDLR, MLH1, MSH2, MSH6, MUTYH, NTHL1, PALB2, PMS2, PTEN, RAD51C, RAD51D, SMAD4, STK11, TP53

**Limitations:** This test may not detect all variants in the genes analyzed. It is designed to be a screening test, not a diagnostic test. This test was done on DNA from saliva collected by research participants. Sample labels were not checked by a health care provider at time of sample collection. Saliva DNA comes mostly from white blood cells. For some people, such as people who have received a bone marrow transplant, this test will not accurately assess inherited risk. If you are concerned that this test may have missed something, you should talk to your doctor about diagnostic genetic testing.

**References**

1. Pritchard CC, Smith C, Salipante SJ, et al. ColoSeq provides comprehensive lynch and polyposis syndrome mutational analysis using massively parallel sequencing. *J Mol Diagn*. 2012;14(4):357-366. doi:10.1016/j.jmoldx.2012.03.002

2. Shirts B, Casadei S, Jacobson A, et al. Clinical Performance of Comprehensive Familial Cancer Screening Using ColoSeq and BROCA. In: ; 2014:699-699.

3. Herman DS, Smith C, Liu C, et al. Efficient Detection of Copy Number Mutations in PMS2 Exons with a Close Homolog. *J Mol Diagn*. 2018;20(4):512-521. doi:10.1016/j.jmoldx.2018.03.010

4. Canedo JR, Miller ST, Myers HF, Sanderson M. Racial and ethnic differences in knowledge and attitudes about genetic testing in the US: Systematic review. *J Genet Couns*. 2019;28(3):587-601. doi:10.1002/jgc4.1078

5. Espenschied C, Pepper J, McFarland RE. What about the guys? An assessment of gender differences in hereditary colorectal cancer testing. *JCO*. 2017;35(4_suppl):537-537. doi:10.1200/JCO.2017.35.4_suppl.537

6. Smith W, Smith K, Sessions W, Evins C, Baker M, Blumer M. An evaluation of gender discrepancies in genetic referrals for BRCA testing for indicated malignancies. *JCO*. 2021;39(15_suppl):10584-10584. doi:10.1200/JCO.2021.39.15_suppl.10584

7. Daly MB, Pal T, Berry MP, et al. Genetic/Familial High-Risk Assessment: Breast, Ovarian, and Pancreatic, Version 2.2021, NCCN Clinical Practice Guidelines in Oncology. *J Natl Compr Canc Netw*. 2021;19(1):77-102. doi:10.6004/jnccn.2021.0001

8. Sturm AC, Knowles JW, Gidding SS, et al. Clinical Genetic Testing for Familial Hypercholesterolemia: JACC Scientific Expert Panel. *J Am Coll Cardiol*. 2018;72(6):662-680. doi:10.1016/j.jacc.2018.05.044

9. Kaphingst K, Facio F, Cheng MR, et al. Effects of informed consent for individual genome sequencing on relevant knowledge. *Clinical Genetics*. 2012;82(5):408-415. doi:10.1111/j.1399-0004.2012.01909.x

10. Fraint A, Ouyang B, Metman LV, et al. Patient Knowledge and Attitudes towards Genetic Testing in Parkinson’s Disease Subjects with Deep Brain Stimulation. *Parkinsons Dis*. 2019;2019:3494609. doi:10.1155/2019/3494609

11. Li M, Bennette CS, Amendola LM, et al. The Feelings About genomiC Testing Results (FACToR) Questionnaire: Development and Preliminary Validation. *Journal of Genetic Counseling*. 2019;28(2):477-490. doi:10.1007/s10897-018-0286-9

12. Eijzenga W, Bleiker EMA, Hahn DEE, et al. Psychosocial Aspects of Hereditary Cancer (PAHC) questionnaire: development and testing of a screening questionnaire for use in clinical cancer genetics. *Psycho-Oncology*. 2014;23(8):862-869. doi:10.1002/pon.3485

13. Sinicrope PS, Vernon SW, Diamond PM, et al. Development and Preliminary Validation of the Cancer Family Impact Scale for Colorectal Cancer. *Genetic Testing*. 2008;12(1):161-169. doi:10.1089/gte.2007.0077
